# Topology-Evolving Image Encryption Algorithm Utilizing 2D Rosenbrock–Schwefel Hyperchaotic Map

**DOI:** 10.3390/e28080926

**Published:** 2026-08-18

**Authors:** Wenjun Song, Hao Shen, Xuncai Zhang, Chengye Zou

**Affiliations:** 1 School of Computer Science and Artificial Intelligence, Zhengzhou University of Light Industry, Zhengzhou 450002, China; songwenjun@zzuli.edu.cn (W.S.); 332405060455@zzuli.edu.cn (H.S.); 2 School of Information Science and Engineering, Yanshan University, Qinhuangdao 066004, China; zouchengye@ysu.edu.cn

**Keywords:** nonlinear dynamics, hyperchaotic map, dynamic topology, security performance

## Abstract

Traditional image encryption methods based on static permutation and diffusion are vulnerable to structural cryptanalysis and often exhibit limited robustness under imperfect communication conditions. To address these issues, this paper proposes a robust topology-evolving image encryption algorithm driven by complex hyperchaotic dynamics for secure visual data transmission. First, a two-dimensional Rosenbrock–Schwefel hyperchaotic map is constructed to generate high-quality pseudorandom sequences for both permutation and diffusion. Based on this map, a bidirectional oscillatory spatial permutation mechanism governed by a dynamic linked-list topology is developed. Unlike fixed-path permutation strategies, the proposed topology continuously evolves with the system state during image traversal, thereby increasing nonlinear path complexity and improving resistance to structural attacks. Furthermore, a plaintext-dependent adaptive diffusion mechanism is designed to enhance sensitivity to plaintext variations and produce a strong global avalanche effect. Experimental results demonstrate that the proposed algorithm achieves favorable encryption performance, with an information entropy of up to 7.9994, a Number of Pixels Change Rate (NPCR) of 99.6076%, and a Unified Average Changing Intensity (UACI) of 33.4683%. In addition, the algorithm maintains good recovery performance under cropping attacks and noise interference, indicating its robustness and applicability for secure image transmission in complex communication environments.

## 1. Introduction

With the rapid advancement of mobile internet, cloud computing, and Internet of Things technologies, digital images have become vital for information transmission and storage and have been extensively applied in fields such as telemedicine, military reconnaissance, commercial information protection, and social networking [1,2,3]. However, as data transmitted through public networks remain extremely susceptible to unlawful interception and tampering, or theft, ensuring the security and privacy of image data has emerged as a shared concern within both academic and industrial circles [4,5].

Compared with textual data, digital images contain large volumes of data, high redundancy, and strong correlations between adjacent pixels. Although conventional ciphers such as AES provide mature security and standardized implementations, their general-purpose designs are not optimized for image-specific statistics and may incur additional computational costs in real-time image applications [6,7]. These characteristics motivate the development of specialized image-encryption methods.

Chaos-based image encryption has been widely regarded as a promising solution for secure image transmission, because of its sensitivity to initial conditions and control parameters, pseudo-random behavior, and favorable ergodicity [8,9]. In such schemes, the design of the chaotic system and its dynamical properties play a central role in determining security. Early studies employed mainly one-dimensional chaotic maps, such as logistic and sine maps. Although these maps are structurally simple and computationally lightweight, they often suffer from limited dynamical complexity, restricted key spaces, and vulnerability to phase-space reconstruction attacks [10,11,12].

To increase dynamic complexity, researchers have developed a variety of high-dimensional chaotic and hyperchaotic systems, including two-dimensional chaotic maps and spatiotemporal chaotic systems [13,14,15]. For example, Cao et al. constructed a memristor-based hyperchaotic system to improve randomness [16]; Teng et al. proposed a two-dimensional chaotic model by cross-coupling the Logistic and sine maps [17]; Gao et al. introduced the two-dimensional Logistic–Rulkov Neuron Map (2D-LRNM), integrating neural dynamics with mathematical chaos [18]; Zhou et al. developed the 2D-ISCC system with strong nonlinear coupling through cross-feedback and function composition [19]; and Nan et al. constructed a two-dimensional Logistic–Cubic cascaded map that broadens the chaotic parameter range and improves ergodicity [20]. Shi et al. proposed a lossless frequency-domain image-encryption scheme combining a 3D exponential hyper-chaotic map with integer lifting wavelet transform for reversible and robust image protection [21]; Yu et al. developed a memristive Hopfield neural-network model with hidden heterogeneous and homogeneous extreme multistability for nonlinear time-series forecasting [22].

More recently, constructing chaotic maps by leveraging optimization test functions has attracted increasing attention. Erkan et al. developed a hyperchaotic system based on the Rosenbrock function [23]. However, owing to the relatively smooth local gradients of the Rosenbrock landscape, the resulting system may exhibit a limited maximum Lyapunov exponent, leaving room to further strengthen the sensitivity to initial conditions. Toktas et al. increased complexity by introducing multimodal functions such as Rastrigin and Griewank [24]. Nevertheless, the extensive use of trigonometric terms and square root operations in their formulation is not favorable for efficient digital implementation and practical engineering deployment. More broadly, many chaos-based designs still suffer from digital degradation, discontinuous chaotic intervals, or narrow effective parameter ranges after discretization, which restricts their applicability in real-world cryptographic scenarios [25,26].

In terms of cryptographic architecture, the permutation–diffusion paradigm has become the dominant framework for image encryption [27]. In this framework, permutation reduces spatial correlations among pixels, whereas diffusion amplifies slight changes in the plaintext into significant variations in the ciphertext. Despite extensive efforts to enhance image encryption by incorporating DNA computing [28,29], compressed sensing [30], cellular automata [31], and neural networks [32], several weaknesses are still frequently observed. First, many permutation mechanisms depend on static topological structures, where the visiting paths remain unchanged across different rounds or different images, which increases exposure to chosen-plaintext attacks [33,34]. Second, conventional diffusion schemes often rely on unidirectional accumulation, which may fail to induce a sufficient global avalanche effect; consequently, robustness can be compromised under noise contamination or data-cropping conditions [35,36].

To overcome these limitations, this paper proposes an image encryption scheme based on a novel two-dimensional Rosenbrock–Schwefel (2D-RS) chaotic map and a dynamic topology-driven permutation–diffusion strategy. The main contributions are summarized as follows.

(1) A novel 2D Rosenbrock–Schwefel hyperchaotic map (2D-RS) is proposed, fusing the coupled valley structure of Rosenbrock with the multipeak oscillation of Schwefel to sustain stable hyperchaos over a wider parameter range and eliminate discontinuous chaotic intervals. This is a substantive innovation in chaotic-source construction.
(2) A bidirectional oscillatory permutation guided by a dynamic linked-list topology is proposed, where chaotic sequences drive the traversal direction and stride in real time, yielding a nonlinear walk over a continuously reconfigured topology and eliminating the periodicity of static paths. This is a substantive innovation in the permutation paradigm.
(3) A state-dependent adaptive cyclic-shift diffusion mechanism is designed, incorporating pixel-level feedback and state-driven bit-level permutation into the classical cyclic-shift XOR framework to enhance the avalanche effect and robustness. This is a structural improvement over existing diffusion methods.

## 2. 2D-RS Map Design

As shown in Table 1, existing two-dimensional chaotic maps usually enhance nonlinear behavior and trajectory complexity through trigonometric functions, fractional structures, variable coupling terms, and multi-parameter control mechanisms. These designs can improve the sensitivity to initial conditions and pseudorandom performance to a certain extent. However, some maps still suffer from relatively simple coupling structures, limited parameter adjustment flexibility, or periodic windows within specific parameter ranges. Therefore, to further enhance the nonlinear perturbation capability, variable coupling strength, and dynamic complexity of two-dimensional chaotic systems, a new two-dimensional chaotic map based on typical optimization functions is constructed in this paper.

### 2.1. Basis Functions for the 2D-RS Map

#### 2.1.1. Rosenbrock Function

The Rosenbrock function is a classical nonconvex optimization test function originally proposed by Rosenbrock in 1960 [39]. It is widely used to assess the search behavior of optimization algorithms under highly variable coupled and ill-conditioned landscapes. Its two-dimensional form is defined as follows:

(1) $$f\left(x,y\right)=100{\left(y-x^{2}\right)}^{2}+{\left(x-1\right)}^{2},$$

**Figure 1:**
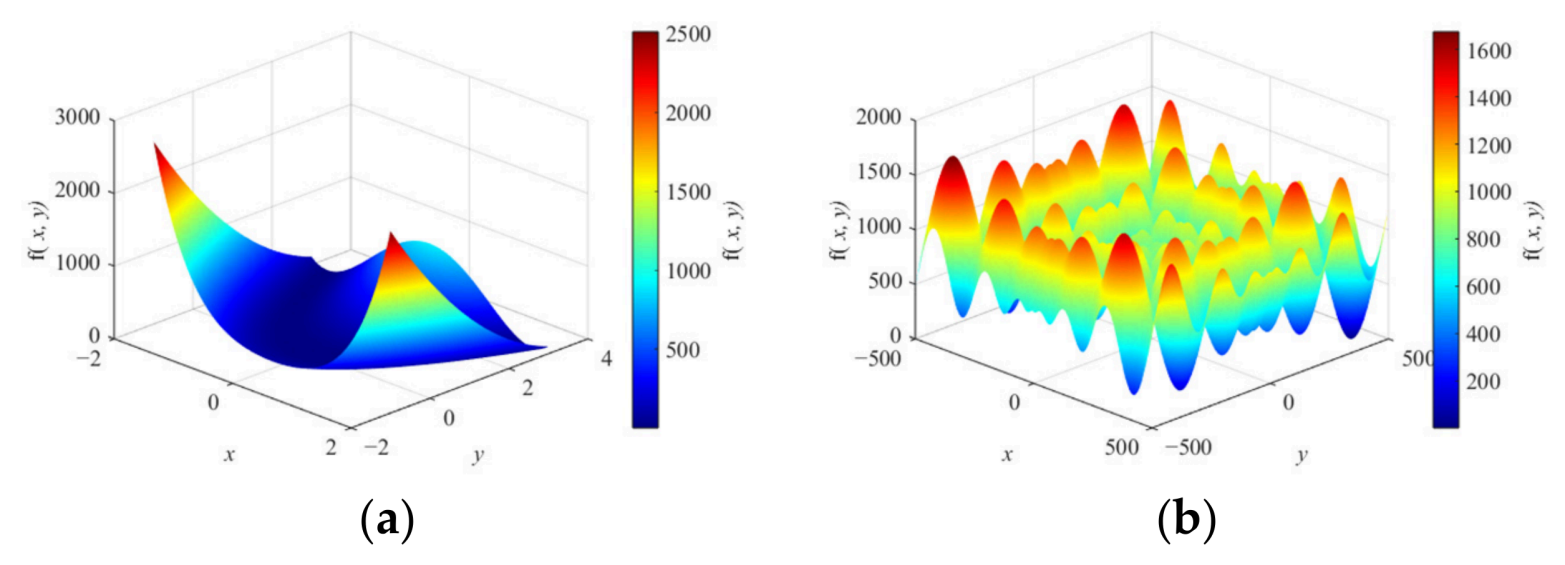
Three-dimensional surface plot of the basis function: (**a**) Rosenbrock and (**b**) Schwefel.

As shown in Figure 1a, the Rosenbrock function results in the formation of a narrow, elongated, and curved valley in the three-dimensional landscape. A key property is the coupling between variables, which leads to markedly anisotropic variation: the function changes slowly along the valley direction but varies sharply in the orthogonal direction. As a result, the function value can respond noticeably to small perturbations of the variables, especially near the valley floor.

From a structural perspective, the pronounced variable coupling and asymmetric valley morphology provide a representative template for constructing mapping models with advanced-order nonlinear interactions.

#### 2.1.2. Schwefel Function

The Schwefel function was originally proposed by Schwefel during research into evolutionary strategies and numerical optimization problems [40], and its typical form is as follows:

(2) $$\begin{array}{c} f\left(x\right)=\sum_{1}^{d}x_{n}\sin(\sqrt{\left|x_{n}\right|}), \end{array}$$

Here, *d* denotes the function dimension.

Unlike the Rosenbrock function, the Schwefel function contains numerous local extrema within its domain and exhibits pronounced oscillatory behavior with a multipeak landscape, as shown in Figure 1b. As the variable range varies, the superposition of oscillatory and linear components produces complex, nonmonotonic changes in the function value. From a structural perspective, the periodic oscillatory term in the Schwefel function offers a representative way to introduce nonlinear perturbations, making it well suited for modeling complex state transitions.

### 2.2. 2D-RS Design

By integrating the structural components of the Rosenbrock and Schwefel functions, we construct the following two-dimensional discrete map:

(3) $$\{\begin{array}{c} x_{n+1}=mod\;(a(x_{n}sin(\sqrt{\left|y_{n}\right|}))-{\mu \left(x_{n}-1\right)}^{3\;}\;,\;\beta ),\\ y_{n+1}=mod\;(b(100{\left(y_{n}-{x_{n}}^{3}\right)}^{3}+{\left(x_{n}-1\right)}^{3})\;,\;\beta ). \end{array}$$

Here, *x_n_* and *y_n_* denote the state variables at the *n*-th iteration; *a*, *b, μ* are control parameters; and *β* is the modulus parameter used to bound the state space. The roles of cubic nonlinearity, cross-variable coupling, and modulo-induced folding are analyzed in Section 2.3.

### 2.3. Analysis on the Chaos Generation Mechanism of the Proposed 2D-RS Map

The chaotic dynamics arise from the joint action of cubic nonlinear amplification, cross-variable coupling, and modulo-induced folding. Their respective effects are further examined through derivative and Lyapunov-exponent analyses.

#### 2.3.1. Mechanism of Local Stretching Enhancement Induced by Cubic Nonlinear Terms

In the proposed map, the quadratic terms of the classical Rosenbrock function are replaced by the cubic nonlinear terms (*y_n_* − *x_n_*^3^)^3^ and (*x_n_* − 1)^3^. Image encryption schemes based on quadratic polynomial hyperchaotic maps and memristor-enhanced polynomial hyperchaotic maps have been previously investigated [41,42].

For a nonlinear residual u satisfying |*u*| > 2/3, the local slope of the cubic term |3*u*^2^| strictly exceeds that of the quadratic term |2*u*|, indicating stronger local stretching over most non-zero residual regions.

To further examine higher odd-order candidates, consider the general odd-order monomial *g_p_*(*u*) = *u^p^* with *p* = 1, 3, 5, 7…, whose mean logarithmic stretching over the unit interval is *L*(*p*) = ln *p* − (*p* − 1). Since *L*′(*p*) = 1/*p* − 1 < 0 for *p* > 1, higher-order odd terms exhibit flatter responses near the origin and may undergo excessive growth before modulo reduction.

Therefore, the cubic term, as the lowest-order nonlinear odd form, provides a reasonable balance between nonlinear stretching, sign preservation, and numerical stability.

#### 2.3.2. Bidirectional Feedback Mechanism Induced by Cross-Variable Coupling

In the proposed map, the argument-dependent term $x_{n}sin(\sqrt{\left|x_{n}\right|})$ in the Schwefel function is revised into the cross-coupling form ${\;x}_{n}sin(\sqrt{\left|y_{n}\right|})$. Consequently, the evolution of *x_n_*_+1_ depends not only on itself but is also directly modulated by *y*_n_. The cross-coupling sensitivity can be reflected by the partial derivative:

(4) $$\frac{\partial x_{n+1}}{\partial y_{n}}=ax_{n}cos\left(\sqrt{\left|y_{n}\right|}\right)\frac{sgn\left(y_{n}\right)}{2\sqrt{\left|y_{n}\right|}},y_{n}\neq 0,$$

Non-zero partial derivatives reveal that subtle variations in *y_n_* can directly perturb *x_n_*_+1_. Meanwhile, *y_n_*_+1_ is correlated with *x_n_* through ${\left(y_{n}-{x_{n}}^{3}\right)}^{3}$, which forms a closed-loop feedback mechanism: *y*_n_ modulates the oscillation of *x_n_*_+1_, and *x_n_* dominates the nonlinear growth of *y_n_*_+1_. This bidirectional coupling renders system trajectories complex and unpredictable, and further enhances the sensitivity to initial conditions.

#### 2.3.3. Bounded Folding Mechanism Induced by Modulus Parameter β

The rapid growth of the cubic nonlinear terms may cause trajectory divergence; the modulus parameter *β* is therefore introduced to confine the state variables to [0, *β*). Similarly, Ding et al. employed a polynomial modulo chaotic map to regulate the state range and the range of Lyapunov exponents [43]. Let *z* = (*x*, *y*) *^T^* denote the state vector, let *F* represent the map before modulo reduction, and let *T_β_* denote the complete map. Here, *J_G_* denotes the Jacobian matrix of a map *G*. The modulo operation makes *T_β_* piecewise smooth. Within each differentiable region away from the folding boundaries, it only subtracts an integer multiple of *β*; hence, *J_Tβ_*(*z*) = *J_F_*(*z*). So the pre-modulo derivative analysis and its local stretching remain valid; at the folding boundaries, branch switching folds the trajectory back into the bounded state space. Consequently, *β* jointly controls the state-space range and the folding frequency, and the two positive Lyapunov exponents reported in Section 3 further confirm the hyperchaotic behavior of the complete map.

## 3. Performance Analysis of the 2D-RS Map

This section evaluates the 2D-RS map in terms of phase-space structure, parameter dependence, initial-condition sensitivity, and sequence complexity using phase portraits, bifurcation diagrams, Lyapunov exponents, entropy measures, correlation dimension, and Kolmogorov entropy.

For a more intuitive assessment, five representative two-dimensional chaotic maps from recent studies are further selected for comparison, including the two-dimensional Chebyshev–Infinite–Collapses map (2D-CICM) [38], the 2D Logistic–Rulkov Neuron Map (2D-LRNM) [19], the 2D ICMIC–Sine and cubic cross-coupled map (2D-ISCC), the 2D Logistic–cubic and ICMIC cross-coupling map (2D-LCIC) [20], and the 2D Sine map and mathematical function map (2D-SFHM) [39]. All the comparative experiments are carried out under the same evaluation settings.

### 3.1. Effect of the Parameters β and μ on Chaotic Dynamics

**Figure 2:**
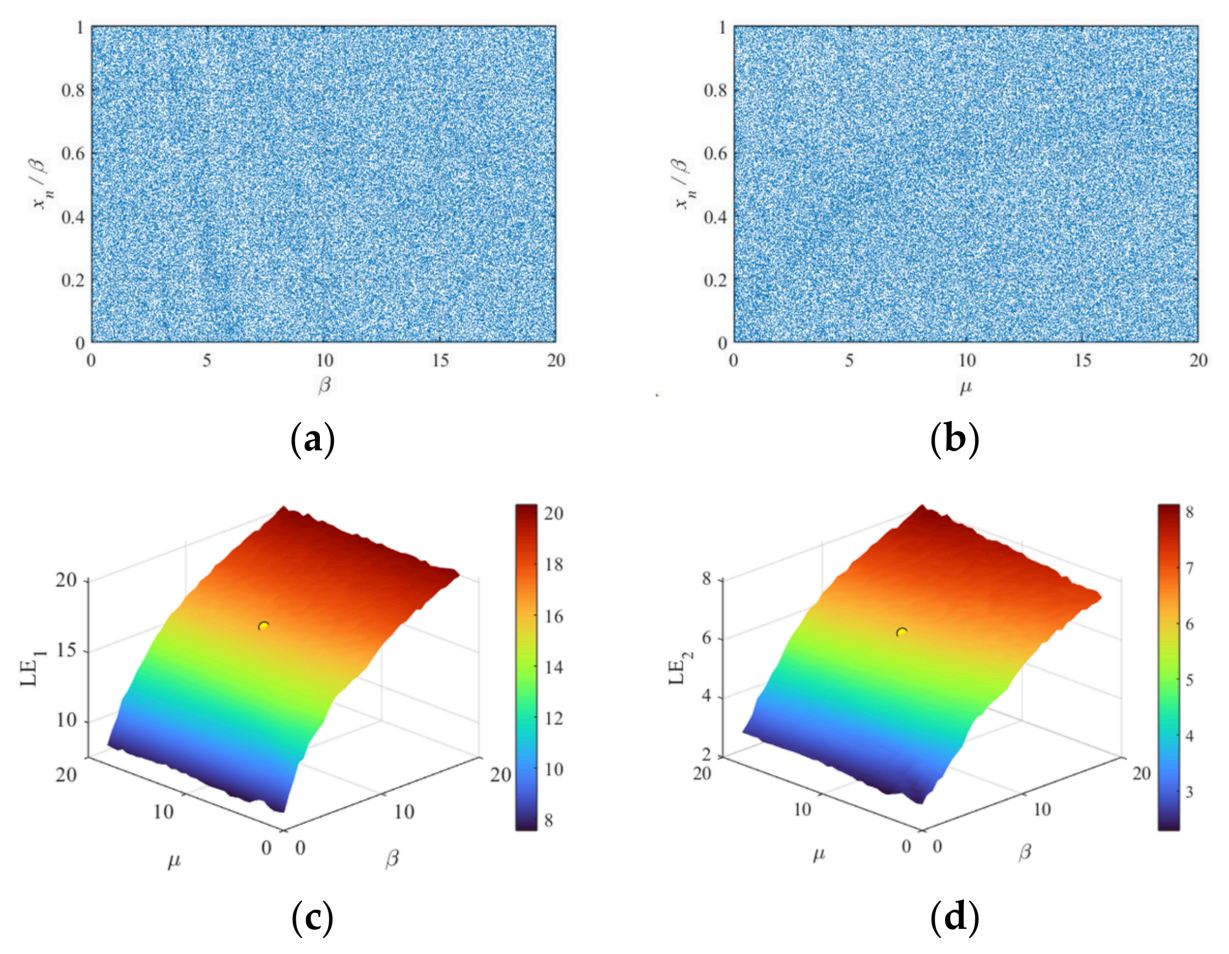
(**a**) Normalized bifurcation diagram with respect to *β.* (**b**) Normalized bifurcation diagram with respect to *μ*. (**c**,**d**) Three-dimensional Lyapunov-exponent surfaces with respect to *μ* and *β*.

To investigate the effects of *β* and *μ* on the system dynamics, parameter scans were performed while keeping the other parameters fixed. Figure 2a and b present the normalized bifurcation diagrams as *β* and *μ* vary, respectively. The results show complex and non-periodic trajectory distributions over the tested ranges. Figure 2c,d further provide the corresponding three-dimensional Lyapunov-exponent analyses. Both Lyapunov exponents remain generally positive across the tested ranges, indicating hyperchaotic behavior. For convenience in the subsequent experiments, *β* = 10 and *μ* = 12 are selected as representative operating points.

### 3.2. Phase Diagram and Attractor Analysis

Phase diagrams provide an intuitive means to examine the dynamic behavior of chaotic systems by visualizing state trajectories and their distribution in phase space [44]. By tracing the iterative trajectories, one can assess phase-space coverage, orbit morphology, and whether low-dimensional attractors or periodic patterns emerge.

**Figure 3:**
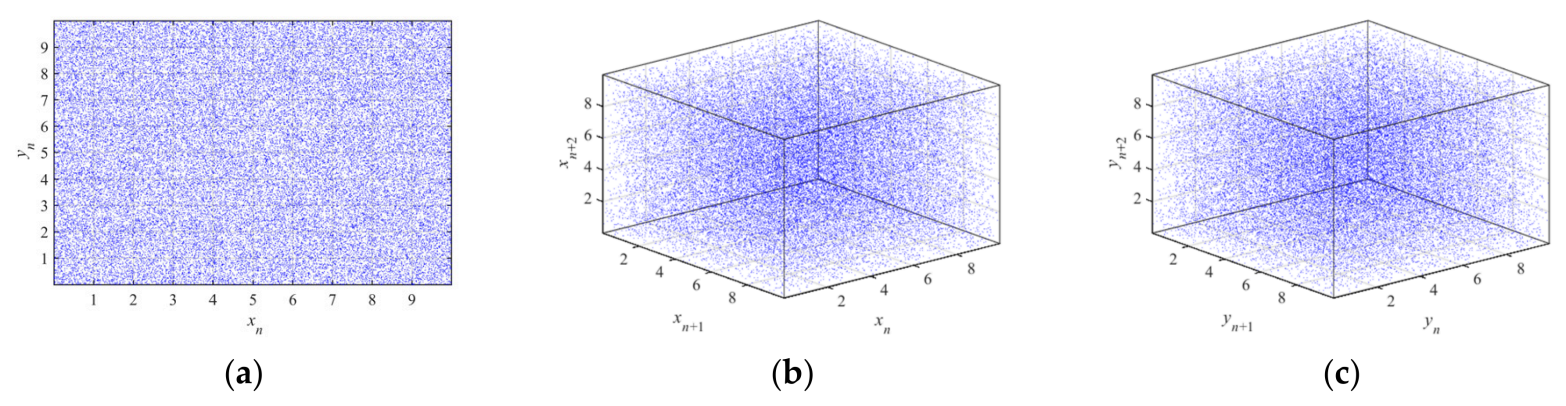
Initial state (*x*_0_, *y*_0_) = (0.6, 0.5), parameter *a* = *b* = 8, *β* = 10: (**a**) phase trajectory of *y_n_* relative to *x_n_*; (**b**) relationship between *x_n_*_+2_ relative to *x_n_*_+1_ and *x_n_*; (**c**) relationship between *y*_(*n*+2)_ and *y_n_*_+1_.

With the control parameters fixed, Figure 3a shows the phase portrait of the proposed 2D-RS map on the *x*-*y* plane. The trajectories exhibit a continuous yet irregular spread across the phase space, repeatedly visiting a wide region without forming evident clusters or simple closed orbits.

To further describe local evolution characteristics, Figure 3b and Figure 3c present three-dimensional embeddings constructed from three consecutive states, namely, (*x_n_*, *x_n_*_+1_, *x_n_*_+2_) and (*y_n_*, *y_n_*_+1_, *y_n_*_+2_), respectively. The resulting trajectories are intricately intertwined in three-dimensional space and do not collapse onto regular surfaces or low-dimensional geometric manifolds, suggesting the absence of obvious low-dimensional attractor structures.

These observations are consistent with the cross-dimensional coupling and oscillatory nonlinear perturbation mechanism introduced in Section 2. From a geometric perspective, the proposed map produces rich trajectories during state updates, which provides a dynamic basis for the subsequent bifurcation analysis, LE evaluation, and complexity assessment.

### 3.3. Bifurcation Diagram Analysis

**Figure 4:**
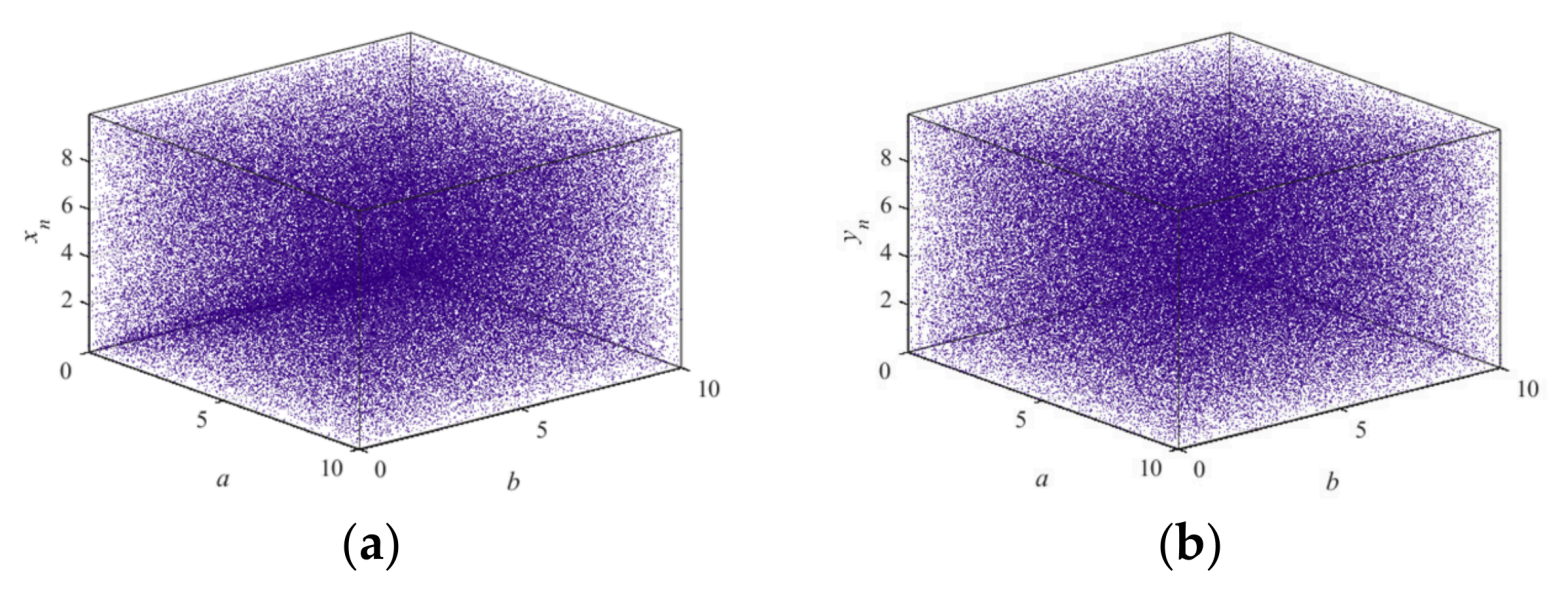
Initial values (*x*_0_, *y*_0_) = (0.6, 0.5) and *β* = 10: bifurcation diagram as control parameters *a* and *b* vary: (**a**) State variable *x_n_*; (**b**) State variable *y_n_*.

Bifurcation analysis examines how a system’s dynamic behavior changes as a control parameter varies and is typically used to evaluate the usable parameter range of chaotic systems. Figure 4a and b show the bifurcation patterns of the state variables *x_n_* and *y_n_* as the control parameters *a* and *b* are varied, respectively. Over a wide parameter interval, both trajectories exhibit dense and irregular distributions, and no evident periodic windows or stable periodic orbits are observed. These results suggest that the 2D-RS map preserves strong chaotic dynamics across a broad range of parameter values.

### 3.4. Lyapunov Exponent (LE) Analysis

LE is a key measure for characterizing chaotic dynamics, quantifying the average exponential rate at which two trajectories starting from nearby initial conditions diverge [45]. For multidimensional systems, a spectrum of LEs typically exists; the presence of at least one positive exponent indicates sensitive dependence on initial conditions and thus chaotic behavior. When more than one exponent is positive, trajectories diverge exponentially along multiple directions, a phenomenon typically referred to as hyperchaotic, which is associated with increased dynamic complexity and unpredictability.

**Figure 5:**
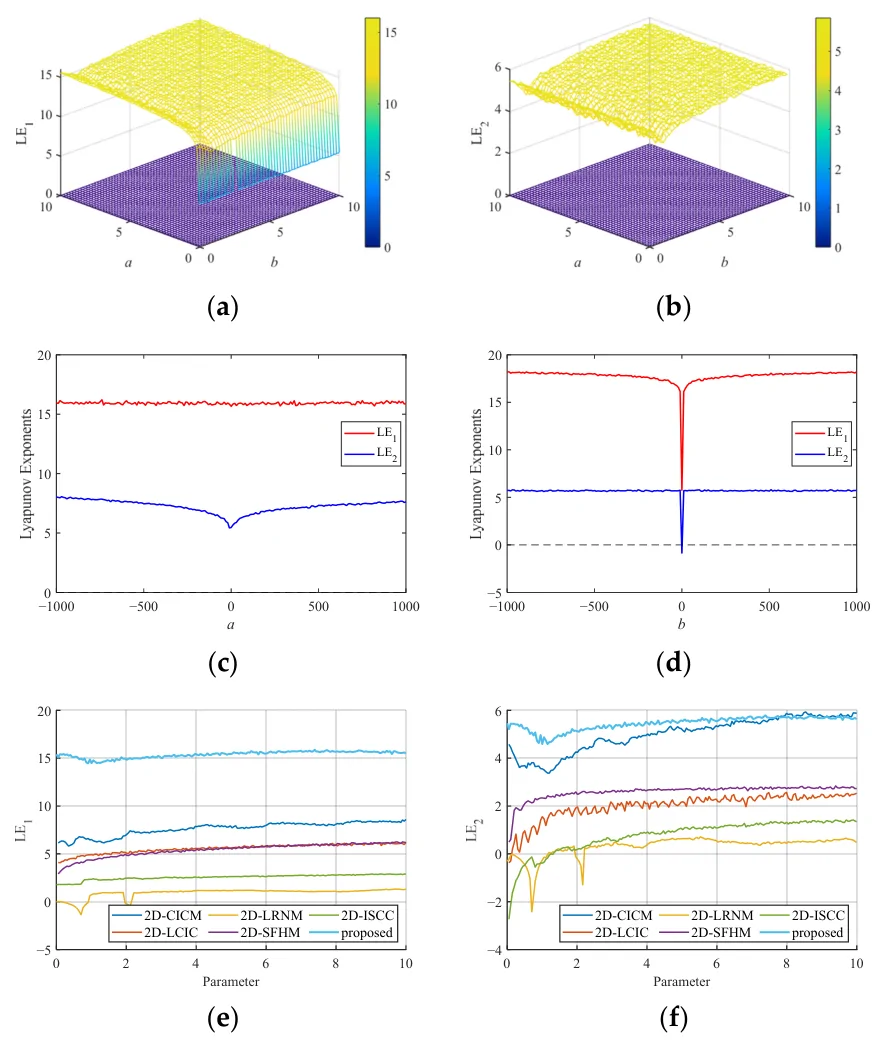
LE analysis: (**a**,**b**) three-dimensional visualization of LE; (**c**,**d**) LE analysis across a broader parameter range; (**e**,**f**) comparative analysis curves of LE versus control parameters.

To assess the proposed 2D-RS map, the two LEs were computed under the specified initial conditions. As shown in Figure 5a,b, both exponents remain positive over a wide range of parameter values, indicating sustained hyperchaotic behavior. To further examine robustness with respect to parameter variation, the scanning ranges of *a* and *b* were extended to (−1000, 1000). The results in Figure 5c,d show that chaotic dynamics persist throughout this enlarged domain without apparent periodic windows, supporting the availability of a large effective key space.

Figure 5e further compares the proposed map with five representative two-dimensional chaotic maps. The 2D-RS map exhibits a consistently positive LE spectrum across the scanned range and yields larger LE magnitudes than the compared maps do in most regions. This suggests that the incorporated cubic advanced-order coupling and cross-oscillatory interaction can amplify small perturbations more effectively and accelerate phase-space divergence, thereby strengthening the unpredictability of the generated sequences.

### 3.5. Information Entropy Analysis

To assess the statistical randomness of sequences generated by the 2D-RS chaotic map, Shannon information entropy is adopted to characterize their probabilistic distribution. Specifically, the continuous-valued sequences produced by the map are first normalized and then quantized into an 8-bit integer space (0–255). The occurrence probability of each symbol is subsequently estimated from the quantized sequence, and the Shannon entropy is computed according to Equation (4):

(5) $$H\left(S\right)=-\sum_{i=0}^{N-1}P\left(s_{i}\right)\log_{2}P\left(s_{i}\right),$$

Here, *S* denotes the information source, and *N* is the number of gray levels (for an 8-bit representation, *N* = 256). Let $s_{i}$ be a symbol (pixel value) and $P\left(s_{i}\right)$ denote its occurrence probability. For an ideal random source, all the symbols appear with equal probability, i.e., $P\left(s_{i}\right)$ = 1/256, in which case the entropy reaches the theoretical maximum of 8 bits. In practice, an entropy value closer to 8 indicates a more uniform distribution and higher randomness, which reduces the risk of statistical information leakage.

**Figure 6:**
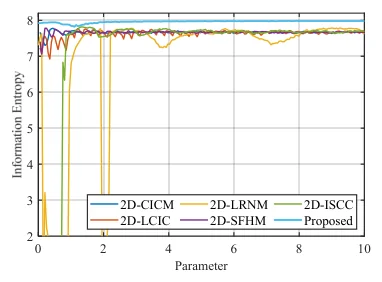
Comparison of Shannon entropy for sequences generated by different chaotic maps.

The results are shown in Figure 6. Compared with the benchmark maps, the proposed 2D-RS map achieves consistently elevated entropy values and is more stable. For several existing systems, the entropy fluctuates noticeably over certain parameter intervals, suggesting degraded ergodicity or the presence of periodic behaviors. In contrast, the 2D-RS map maintains robust and stable entropy across the scanned range, without evident clustering in symbol probabilities. These observations indicate that the 2D-RS map can continuously produce pseudorandom sequences with high uncertainty, thereby improving resistance to statistical analysis attacks.

### 3.6. Sequence Complexity Analysis

#### 3.6.1. Sample Entropy Analysis

Sample entropy (SE) is widely used to quantify the complexity and irregularity of a time series [46]. Larger SE values generally indicate weaker regularity and less predictability, implying greater randomness and dynamic complexity.

**Figure 7:**
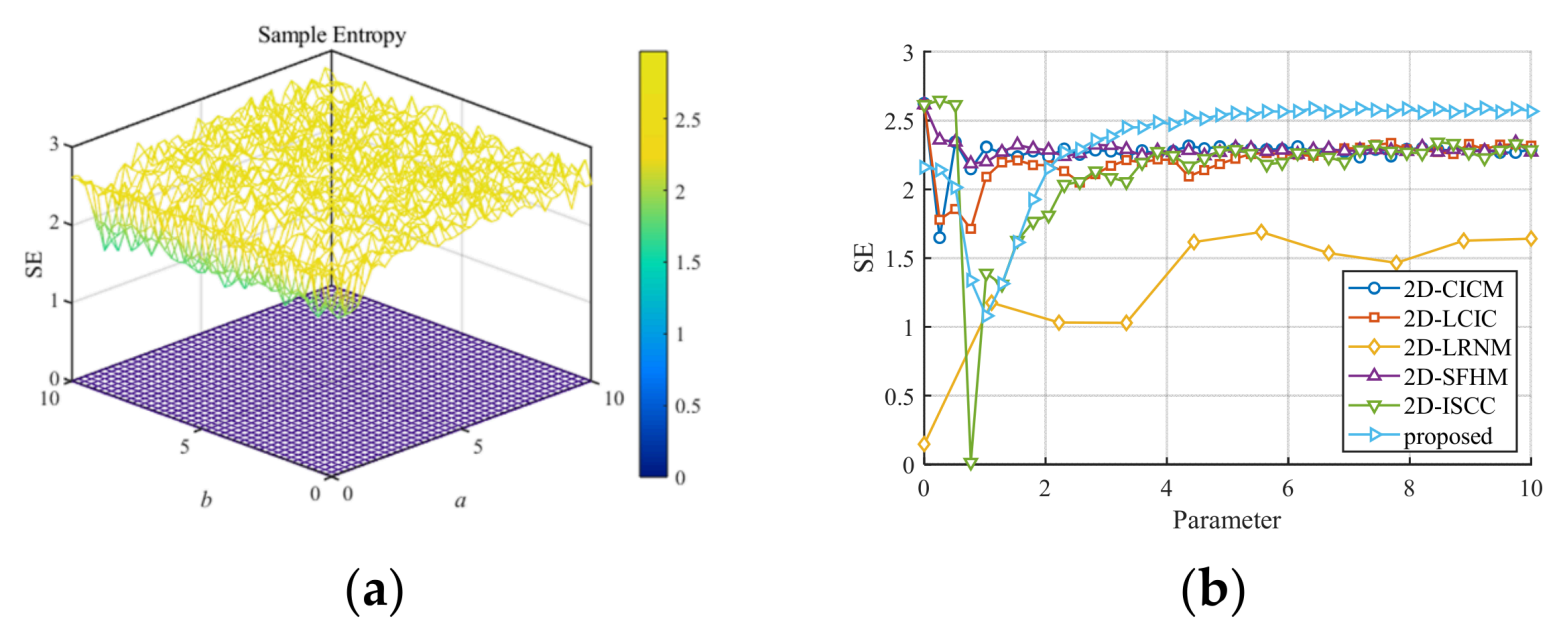
SE analysis: (**a**) three-dimensional visualization of SE; (**b**) comparative analysis curve of SE versus control parameters.

Figure 7a shows the SE of sequences generated by the proposed 2D-RS map as the control parameters vary. The SE remains persistently elevated and fluctuates little across the scanned range, suggesting that the generated sequences retain robust complexity under different parameter settings. Figure 7b further compares the 2D-RS map with five representative two-dimensional chaotic maps. In most parameter intervals, the 2D-RS map yields higher SE values than the compared systems do, indicating enhanced sequence irregularity.

This behavior can be attributed to the joint effect of the advanced-order nonlinear terms and the oscillatory component embedded in the 2D-RS map. Their interaction makes it difficult for the system to settle into repetitive patterns over multiple time scales, supporting the suitability of the proposed map for keystream generation from a complexity perspective.

#### 3.6.2. Kolmogorov Entropy Analysis

Kolmogorov entropy (KE) measures the rate of information production during state evolution and is typically used to characterize the unpredictability of a dynamical system [47]. For nonlinear systems, a positive KE implies sustained information generation, and larger KE values generally indicate that future states are more difficult to predict.

**Figure 8:**
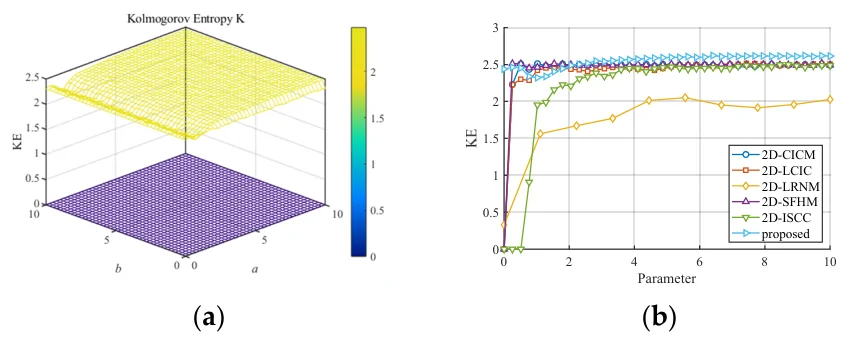
KE analysis: (**a**) three-dimensional visualization of KE; (**b**) comparative analysis curve of KE versus control parameters.

In our experiments, the control parameters *a* and *b* were scanned within the interval (0, 10). Figure 8a shows the three-dimensional distribution of KE for the proposed 2D-RS map. The KE remains robust across the scanned range, suggesting high unpredictability in the system dynamics. The comparative results in Figure 8b further indicate that the 2D-RS map achieves greater overall KE levels than the benchmark maps do, implying a faster information generation rate. As a consequence, the sequences produced by the 2D-RS map are statistically closer to an ideal random source, which strengthens resistance to prediction-based attacks and statistical analysis.

#### 3.6.3. Correlation Dimension Analysis

The correlation dimension (CD) is widely used to characterize the fractal structure of trajectories reconstructed in phase space and can help distinguish deterministic chaos from purely stochastic processes. In general, a finite, noninteger CD indicates fractal geometry and is commonly regarded as evidence of deterministic chaotic dynamics. A larger CD suggests that trajectories occupy a greater effective dimension in phase space, reflecting richer dynamical structures.

**Figure 9:**
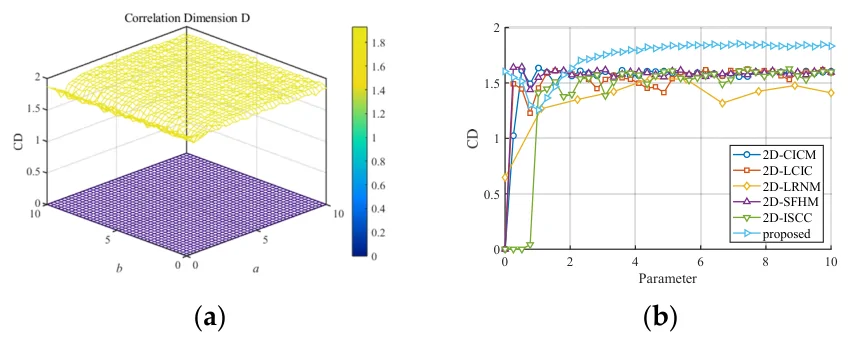
CD analysis: (**a**) three-dimensional visualization of CD; (**b**) comparative analysis curve of CD versus control parameters.

As shown in Figure 9a, the proposed 2D-RS map maintains a stable noninteger CD over the scanned parameter range, providing further support for its deterministic chaotic behavior. The comparison in Figure 9b shows that the 2D-RS map yields higher CD values than several benchmark two-dimensional chaotic maps do, implying stronger phase-space filling and more intricate geometric structures. From a fractal-geometry perspective, these results corroborate the advantage of the 2D-RS map in terms of dynamical complexity.

### 3.7. Sensitivity Analysis of Initial Conditions and Control Parameters

Sensitivity to initial conditions is a hallmark of chaotic dynamics, and one of the main reasons for this is that chaotic maps are considered suitable as random sources in cryptography.

**Figure 10:**
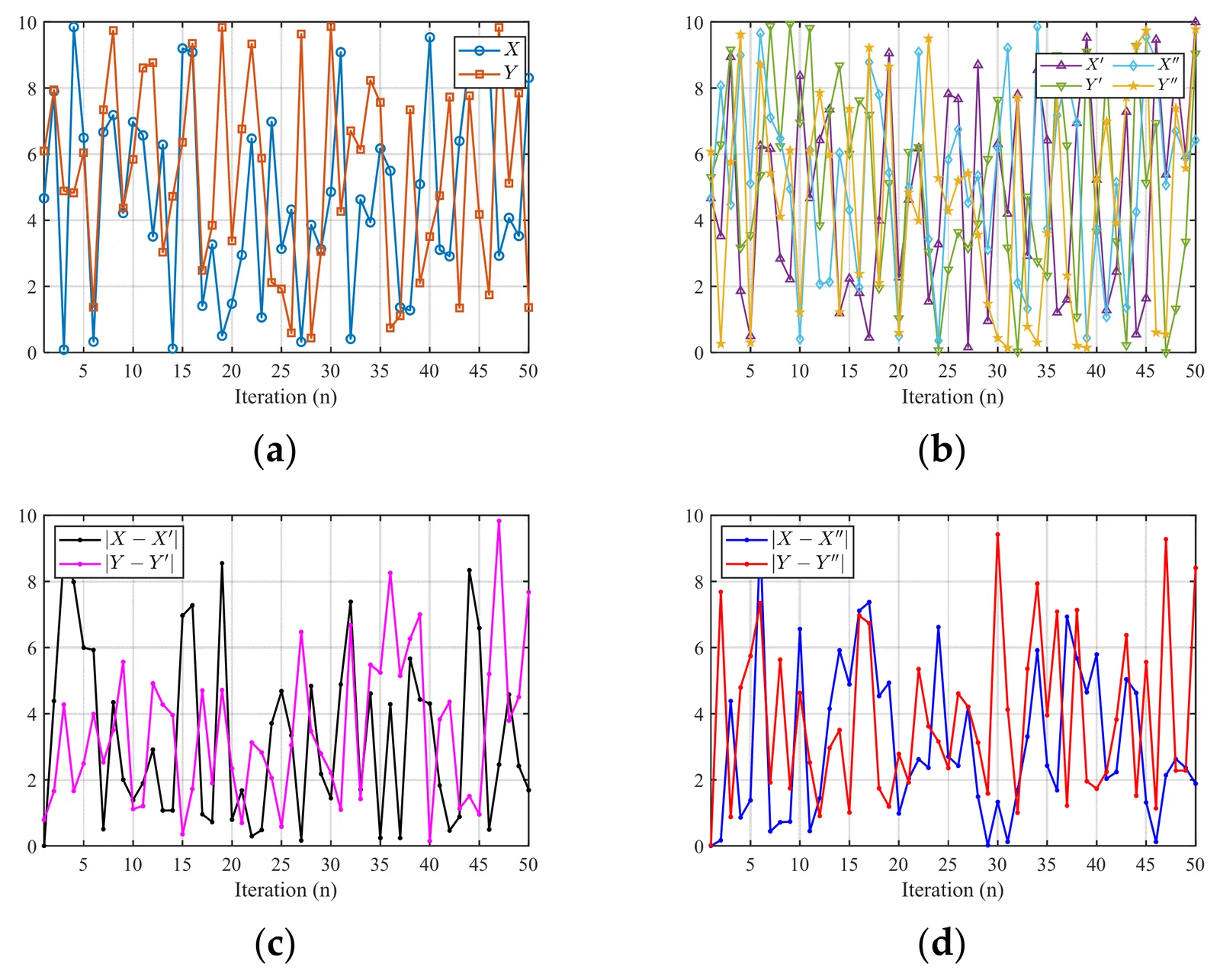
Sensitivity analysis of the proposed 2D-RS map to initial conditions and control parameters: (**a**) reference sequences *X* and *Y* generated from the original initial conditions; (**b**) perturbed sequences *X′* and *Y′* obtained by changing the initial conditions, together with sequences *X″* and *Y″* obtained by perturbing the control parameters; (**c**) absolute differences |*X*−*X′*| and |*Y*−*Y′*| caused by the initial-condition perturbation; (**d**) absolute differences |*X*−*X*″| and |*Y*−*Y*″| caused by the control-parameter perturbation.

To examine the initial-condition sensitivity of the proposed 2D-RS map, two types of perturbations are considered: perturbations to the initial state and perturbations to the control parameters. First, with the control parameters fixed, a reference sequence {*X*, *Y*} is generated from a chosen initial state. A perturbation of 10^−10^ is then applied to the initial condition to produce the perturbed sequence {*X*′, *Y*′}. The corresponding phase trajectories and the differences during the early iterations are shown in Figure 10a–c.

Next, to evaluate parameter sensitivity, the same perturbation magnitude is introduced to the control parameters while keeping the initial state unchanged, yielding {*X*″, *Y*″}. Its phase trajectory and divergence relative to the reference sequence are presented in Figure 10b and Figure 10d, respectively.

As evidenced in Figure 10, very small changes in either the initial state or the control parameters lead to rapid trajectory separation after only a few iterations. This confirms that the 2D-RS map is highly sensitive to both initial conditions and parameters, which is consistent with typical chaotic behavior. The observed divergence behavior supports the use of the proposed map as a core component for keystream generation and for the permutation–diffusion process in image encryption.

### 3.8. NIST Test

The NIST SP 800-22 statistical test suite was employed to evaluate the randomness of the generated sequences. In the experiments, binary sequences of 10^6^ bits generated from chaotic states *x* and *y* were tested with the significance level α set to 0.01.

As shown in Table 2, all *p* values exceeded 0.01. These results confirm that the proposed 2D-RS map exhibits excellent pseudorandomness and unpredictability, making it suitable for secure image encryption.

## 4. Pixel Permutation and Diffusion Strategy

To address the redundancy and high interpixel correlation inherent in image data, we develop an encryption framework in which pixel permutation and diffusion are tightly coupled. Unlike conventional designs that execute two largely independent stages in a fixed order, the proposed scheme first performs a dynamic topology-driven traversal to permute pixel positions in a nonlinear manner over the entire image. It then applies an adaptive bidirectional diffusion procedure to further conceal pixel intensities. By coupling spatial reordering with value transformation, the proposed design suppresses recognizable plaintext patterns in both the spatial and statistical domains, thereby strengthening resistance to statistical analysis and differential attacks.

### 4.1. Dynamic Topology-Based Bidirectional Oscillatory Traversal Permutation

Most existing pixel permutation methods operate on static topologies and use boundary-handling rules such as modulo wrapping. We instead organize the pixels into a doubly linked list and use chaotic direction and stride controls; after each visit, the node is removed and its neighbors are reconnected, causing the topology and subsequent paths to evolve dynamically.

Let the original image be reshaped into a one-dimensional pixel sequence $P=\left(p_{1},p_{2},p_{3},\ldots ,p_{N}\right)$ in row-major order, where *N* is the total number of pixels. A doubly linked list is then built over this sequence. Each node stores one pixel and maintains pointers with its predecessor and successor, forming a linear topology with explicit head and tail boundaries.

The permutation process is controlled by two external sequences: a step-size sequence $A=\left(a_{1},a_{2},a_{3},\ldots ,a_{N}\right)$ and a direction control sequence $B=\left(b_{1},b_{2},b_{3},\ldots ,b_{N}\right)$. Sequence *A* specifies how many nodes the agent moves at each iteration, while the parity of *B* determines whether the current moving direction is preserved or reversed. Both *A* and *B* are obtained by quantifying the pseudorandom sequences generated by the proposed 2D-RS chaotic map.

To avoid inefficient traversal in high-resolution images, excessively large strides are not permitted. The maximum stride length is set to $l=\left(\left\lfloor \sqrt{N}/2\right\rfloor \right)$. The actual step size is dynamically generated within the range [1, *l*], allowing the traversal range to scale with the image size while maintaining path diversity and limiting computational cost.

At the beginning of permutation, the agent is placed at the head of the linked list. In each iteration, it moves according to the current step size *a_i_*. Let *m_t_* denote the number of nodes remaining in the linked list at iteration *t.* Since the currently visited node is deleted after each iteration, a repeated complete traversal state after *T* > 0 iterations would require the list length to remain unchanged; however, $m_{t+T}=m_{t}-T\neq m_{t}$. Therefore, although the rebound rule produces bounded oscillatory motion, it cannot form a nontrivial cycle in the continuously evolving linked-list topology. The traversal path is further affected by the varying step sizes, direction controls, and list rewiring.

The doubly linked list avoids global sorting and supports constant-time node deletion, thereby maintaining linear space scalability; however, with a maximum traversal length of $l=\left(\left\lfloor \sqrt{N}/2\right\rfloor \right)$, the permutation stage has a worst-case complexity of *O*(*N*^3/2^) and may become the main efficiency bottleneck for very high-resolution images.

**Figure 11:**
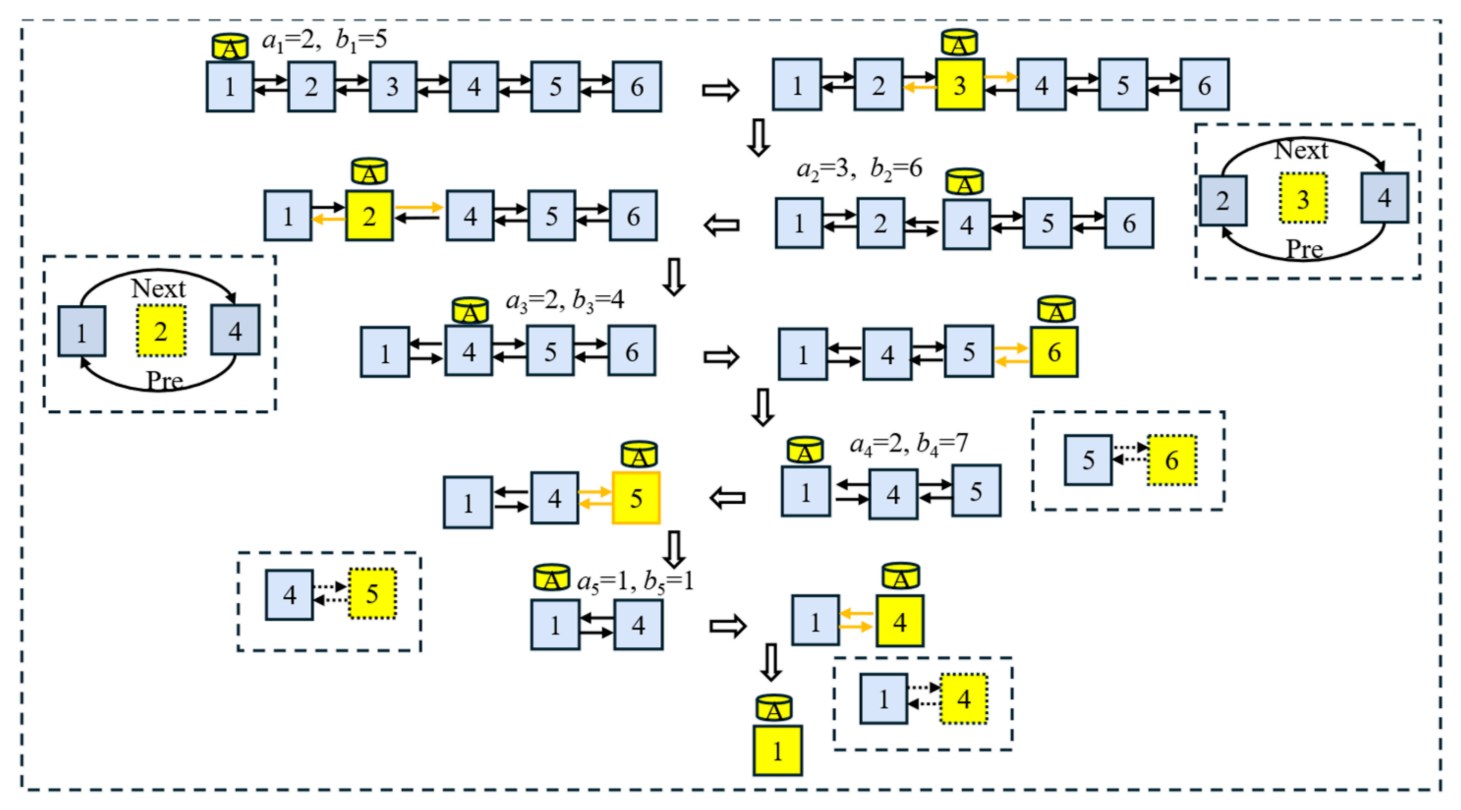
Schematic diagram of topological permutation.

Figure 11 provides a simplified six-node example to illustrate the key idea of dynamic topology evolution. As nodes are removed and the list is rewired, the visiting order becomes highly nonlinear and nonperiodic, highlighting the effectiveness of the proposed strategy in disrupting spatial relationships in the image.

### 4.2. A Bidirectional Diffusion Strategy Based on an Adaptive Dynamic Shift

Many existing diffusion schemes use fixed or predefined shift rules and weak plaintext coupling [48]. Our method generates the shift amount from neighboring ciphertext values and combines forward and reverse scans, enabling more global propagation of plaintext perturbations.

#### 4.2.1. Forward Diffusion

**Figure 12:**
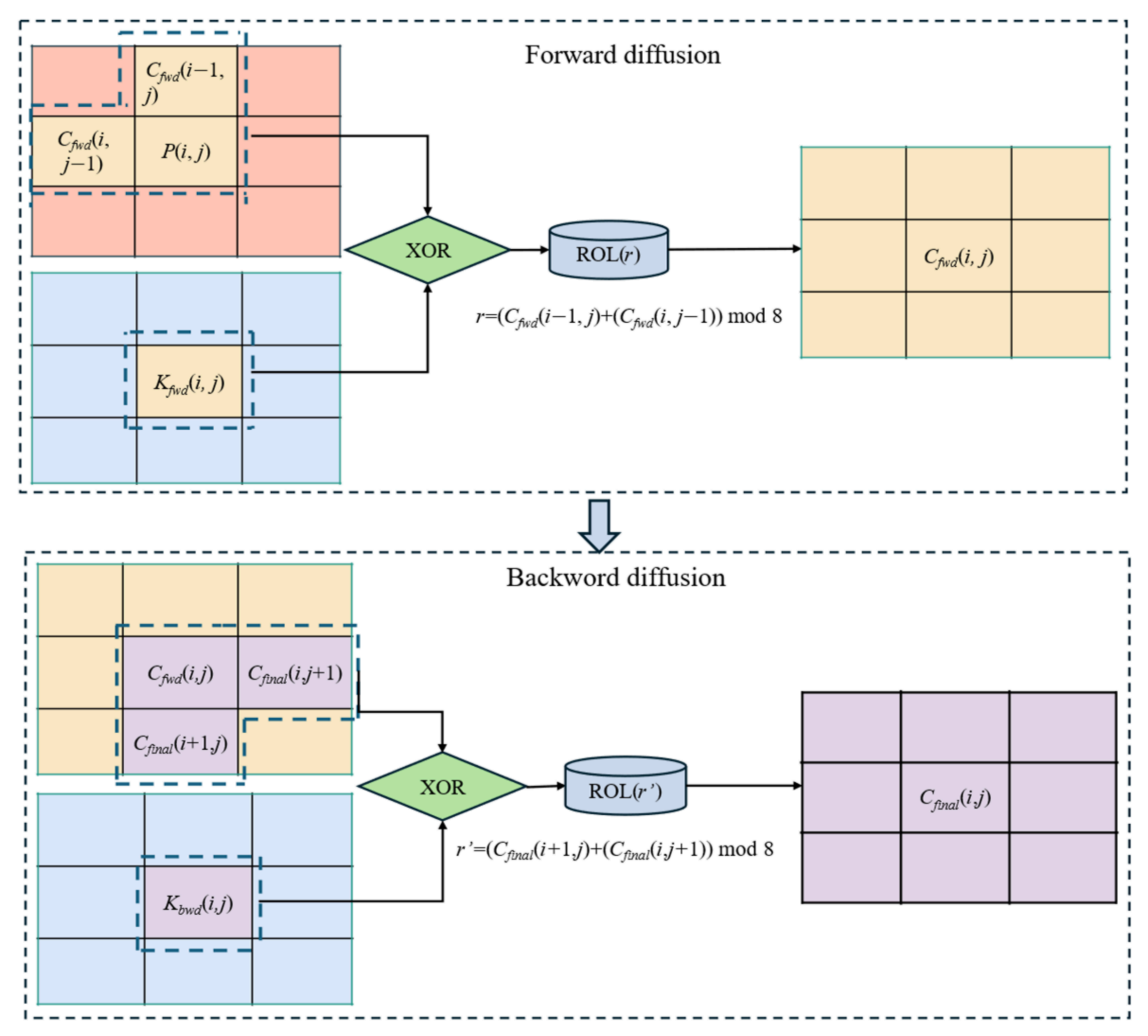
Schematic diagram of bidirectional diffusion with adaptive dynamic shift.

In the forward diffusion stage, pixels are processed in raster order (left-to-right, top-to-bottom). As illustrated in Figure 12, encryption of the current pixel *P* (*i*, *j*) depends not only on the corresponding keystream mask *K_fwd_* (*i*, *j*) but also on two already encrypted neighbors, namely, the upper pixel *C_fwd_* (*i* − 1, *j*) and the left pixel *C_fwd_* (*i*, *j* − 1), through a coupled XOR operation. This chaining mechanism establishes joint horizontal and vertical dependencies, which facilitate the propagation of local changes during diffusion.

Subsequently, a dynamic cyclic-shift operation is applied. The shift amount is computed on the fly from the ciphertext values of the already encrypted neighbors (i.e., the upper and left pixels) of the current position so that the shift is adaptively driven by the local ciphertext context:

(6) $$r(i,j)=\left(C_{fwd}\left(i-1,j\right)+C_{fwd}\left(i,j-1\right)\right)\;mod\;8$$

The XOR result is then cyclically left-shifted by *r* (*i, j*) positions to produce the intermediate ciphertext *C_fwd_* (*i*, *j*). This operation introduces a bit-level state-dependent bit-level permutation, where the shift rule adapts to the local ciphertext context, thereby increasing diffusion sensitivity to nearby variations.

#### 4.2.2. Reverse Diffusion

To address the directional limitation of purely forward diffusion, a reverse diffusion stage is performed immediately after the forward pass using a reverse raster scan from bottom-right to top-left. In this stage, the ciphertext produced by forward diffusion is taken as the input, and the same dynamic coupling and cyclic-shift operations are applied in the opposite traversal direction.

By combining forward and reverse passes, the proposed bidirectional diffusion architecture enables local changes at any position to propagate more thoroughly across the image, promoting a global avalanche effect. In practice, even a minute plaintext perturbation can trigger widespread changes in the ciphertext matrix, which strengthens resistance to differential and statistical attacks.

## 5. Encryption Scheme

**Figure 13:**
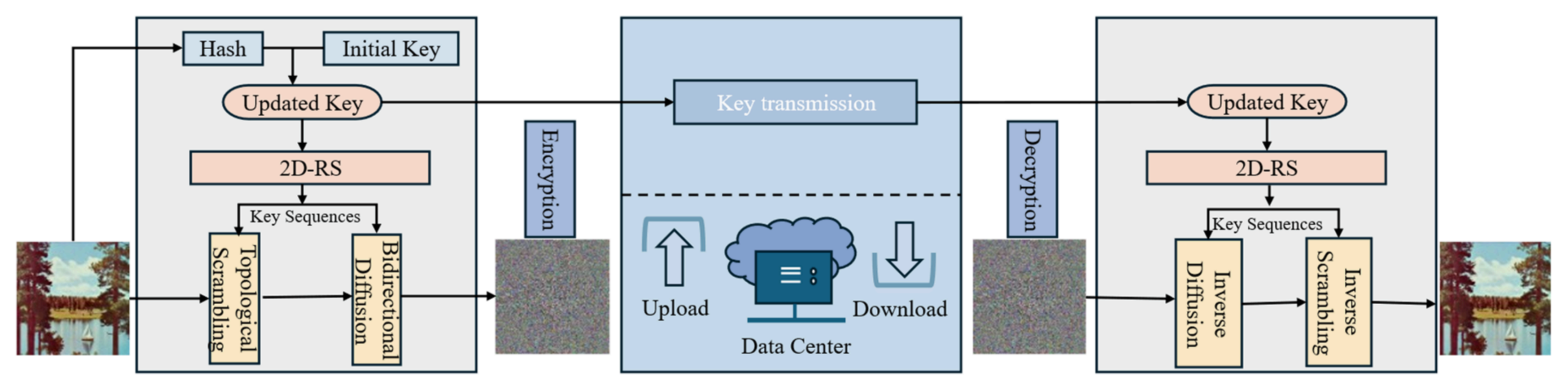
Overall encryption and decryption flowchart.

Building on the proposed 2D-RS chaotic map and the associated permutation–diffusion strategy, we develop a complete image encryption algorithm, with the overall workflow shown in Figure 13. The scheme consists of three main modules: plaintext-related key generation, dynamic topology-driven pixel permutation, and adaptive bidirectional diffusion. All the modules are driven by chaotic sequences, which tightly couple the data and control layers.

### 5.1. Key Sequence Generation

In chaos-based cryptosystems, the quality of the keystream and its binding to the plaintext play a central role in overall security. In the proposed scheme, SHA-512 is combined with the 2D-RS map to generate pseudorandom sequences for the subsequent permutation and diffusion stages.

Let the plaintext image *P* have dimensions *H* × *W* × *C*. Given the external secret parameters, including the initial state $\left\{x_{0},y_{0}\right\}$, the control parameters *a* and *b*, and the modulus factor *β*, a plaintext-dependent keying mechanism is introduced to mitigate chosen-plaintext attacks. Specifically, the 512-bit SHA-512 hash of *P* is computed and denoted by *H*, which is then divided into 64 bytes: *H* = [*h*_1_, *h*_2_, *h*_3_…, *h*_64_], where *h_i_* ∈ [0, 255] for *i* = 1, 2, 3,…,64. In Equation (7), the SHA-512 digest is first represented as 64 bytes and divided into eight consecutive byte groups. The sums of adjacent groups are combined using bitwise XOR, where ⊕ denotes the bitwise XOR operation. The combined values are then divided by 2^10^ to scale their numerical range, followed by the addition of the corresponding *x*_0_ or *y*_0_. Finally, modulo 1 is applied to normalize the results to [0,1), producing two initial-state pairs, $\left\{x_{0}^{1},y_{0}^{1}\right\}$ and $\left\{x_{0}^{2},y_{0}^{2}\right\}$, which are used to drive the generation of the required chaotic sequences.

(7) $$\{\begin{array}{l} x_{0}^{1}=\left(\frac{\sum_{i=1}^{8}h_{i}⊕\sum_{i=9}^{16}h_{i}}{2^{10}}+x_{0}\right)mod1,\\ y_{0}^{1}=\left(\frac{\sum_{i=17}^{24}h_{i}⊕\sum_{i=25}^{32}h_{i}}{2^{10}}+y_{0}\right)mod1,\\ x_{0}^{2}=\left(\frac{\sum_{i=33}^{40}h_{i}⊕\sum_{i=41}^{48}h_{i}}{2^{10}}+x_{0}\right)mod1,\\ y_{0}^{2}=\left(\frac{\sum_{i=49}^{56}h_{i}⊕\sum_{i=57}^{64}h_{i}}{2^{10}}+y_{0}\right)mod1. \end{array}$$

With the updated key parameters, the 2D-RS map is iterated for *Q* + *N* steps, where *N* = *H* × *W* × *C*. After discarding the first *Q* transient iterations, two pairs of chaotic state sequences of length *N* are obtained: *X*_1_ and *Y*_1_ generated from $\left\{x_{0}^{1},y_{0}^{1},a,b,\mu ,\beta \right\}$ and *X*_2_ and *Y*_2_ generated from $\left\{x_{0}^{2},y_{0}^{2},a,b,\mu ,\beta \right\}$. These sequences are subsequently used to drive the permutation and diffusion stages.

### 5.2. Pixel Permutation

This section details the implementation of the bidirectional oscillatory traversal permutation strategy introduced in Section 4.1. By mapping and quantizing the sequences generated by the chaotic map, the abstract traversal mechanism is converted into an operation that can be directly applied to pixel data, thereby enabling nonlinear reordering of pixel positions. The overall permutation procedure consists of two stages: global index permutation and local traversal permutation under a dynamic topology.

Given a one-dimensional pixel sequence $P=\left(p_{1},p_{2},p_{3},\ldots ,p_{N}\right)$, where *N* is the total number of pixels, the chaotic sequence *X*_1_ generated by the 2D-RS map is first sorted in ascending order to obtain an index sequence *I_S_*. The pixel sequence is then globally permuted according to *I_S_*, yielding a prepermuted sequence *P*_2_.

(8) $$\left[\sim ,I_{S}\right]=sort\left(X_{1}\right)$$

(9) $$P_{2}=P_{1}\left(I_{S}\left(k\right)\right),k=1,2,3,\ldots ,N$$

After the global index permutation, the dynamic topology-driven traversal strategy described in Section 4.1 is applied to further suppress any residual local spatial correlation. To drive this traversal process, the continuous-valued chaotic outputs are converted into discrete control parameters. Specifically, the chaotic sequences *X*_1_ and *Y*_1_ are quantized to generate the step-size sequence *L* and the direction-control sequence *D*, respectively.

(10) $$\{\begin{array}{c} L=\left\lfloor Y_{1}\times 10^{3}\right\rfloor \;mod\;l+1,\\ D=\left\lfloor {(X}_{1}+Y_{1})\times 10^{2}\right\rfloor . \end{array}$$

Here, *l* denotes the maximum allowable stride, which limits the movement range in each traversal step and provides a practical trade-off between path randomness and computational efficiency.

Using these control sequences, the prepermuted sequence *P*_2_ is organized into a doubly linked list. Node visiting and deletion are then carried out according to the traversal rules described in Section 4.1. As nodes are removed and the list is rewired, the agent’s accessible paths change continuously, producing a highly nonlinear and nonperiodic visiting order. Pixel values are output sequentially following this visiting order, resulting in the permuted sequence *S′*.

### 5.3. Pixel Diffusion

This section describes the implementation of the bidirectional adaptive diffusion strategy introduced in Section 4.2. Chaotic sequences are first processed to construct diffusion masks that match the image dimensions. Together with neighborhood ciphertext information, these masks enable pixel-level feedback during diffusion, leading to thorough concealment of pixel intensities.

To support diffusion in both the forward and reverse directions, the chaotic sequences *X*_2_ and *Y*_2_ generated in Section 5.1 are transformed into diffusion-mask matrices for the forward and backward scans, respectively. The corresponding mask matrices *K*_1_ and *K*_2_ are defined as follows:

(11) $$\{\begin{array}{c} K_{1}=\left\lfloor X_{2}\times 10^{5}\right\rfloor \;mod\;256,\\ K_{2}=\left\lfloor Y_{2}\times 10^{5}\right\rfloor \;mod\;256. \end{array}$$

During forward diffusion, the encryption of the current pixel *C*_1_ (*i*, *j*) is jointly determined by the diffusion mask *K*_1_ and an adaptive cyclic-shift operation driven by neighboring ciphertext values. For the pixel at position (*i*, *j*), the update rule is defined as follows:

(12) $$C_{1}\left(i,j\right)=ROL\left(S^{\prime }\left(i,j\right)⊕K_{1}\left(i,j\right)⊕C_{1}\left(i-1,j\right)⊕C_{1}\left(i,j-1\right),r\right)$$

(13) $$r={(C}_{1}\left(i-1,j\right)+C_{1}\left(i,j-1\right))\;mod\;8$$

Here, ⊕ denotes the XOR operation, and ROL (*x*, *r)* represents a cyclic left shift in *x* by *r* bits. Subsequently, the intermediate ciphertext *C*_1_ is diffused again using mask *K*_2_ under a reverse scanning order so that the avalanche effect can propagate globally across the entire ciphertext image.

(14) $$C\left(i,j\right)={ROL(C}_{1}\left(i,j\right)⊕K_{2}\left(i,j\right)⊕C\left(i+1,j\right)⊕C\left(i,j+1\right),r^{\prime })$$

(15) $$r^{\prime }=\left(C\left(i+1,j\right)+C\left(i,j+1\right)\right)\;mod\;8$$

### 5.4. Complete Encryption Procedure

The complete image-encryption procedure is summarized as follows.

**Step 1:** Input the plaintext image *P* and compute its SHA-512 hash digest. In accordance with Equation (7), the digest is combined with the secret initial parameters to obtain two independent initial states, $\left\{x_{0}^{1},y_{0}^{1}\right\}$ and $\left\{x_{0}^{2},y_{0}^{2}\right\}$.

**Step 2:** The updated initial states are used to iterate the 2D-RS map to generate two pairs of chaotic sequences. The sequences *X*_1_ and *Y*_1_ are used for the permutation stage, whereas *X*_2_ and *Y*_2_ are used for the diffusion stage.

**Step 3:** Compute the step-size sequence *L* and direction-control sequence *D* for the dynamic linked-list traversal using *X*_1_ and *Y*_1_ according to Equation (10). The diffusion masks *K*_1_ and *K*_2_ are computed from *X*_2_ and *Y*_2_ according to Equation (11).

**Step 4:** Flatten *P* into a one-dimensional sequence *P*_1_. Sort *X*_1_ in ascending order to obtain the index sequence, and permute *P*_1_ accordingly to produce the prepermuted sequence *P*_2_.

**Step 5:** Convert *P*_2_ into a doubly linked-list structure. Guided by *L* and *D*, an agent performs a nonlinear walk on the list and repeatedly executes node visiting and deletion to generate the final permuted sequence. The result is then reshaped into a two-dimensional matrix, denoted as *P*_3_.

**Step 6:** To handle boundary neighborhoods during diffusion, a zero-valued padding border is added around *P*_3_ (only for diffusion computation and not included in the final ciphertext). First, forward dynamic cyclic-shift diffusion is performed using mask *K*_1_ according to Equations (12) and (13). Afterward, reverse dynamic cyclic-shift diffusion is performed using mask *K*_2_ according to Equations (14) and (15). After the padded borders are removed, the final ciphertext image *C* is obtained.

Since both permutation and diffusion operations are reversible, decryption can be achieved by executing the above steps in reverse order.

## 6. Experimental Results and Analysis

In this section, the proposed image-encryption scheme is evaluated through simulation experiments, and its effectiveness and reliability are assessed from multiple aspects, including visual quality, security, and computational performance. All the experiments were carried out on a Windows 10 platform using MATLAB R2021b. Unless otherwise stated, the initial state and control parameters of the 2D-RS map were fixed at $\left\{x_{0},y_{0},a,b,\mu ,\beta \right\}=\left\{0.6,0.5,8,8,12,10\right\}$ to ensure experimental consistency and reproducibility.

### 6.1. Visual Analysis

**Figure 14:**
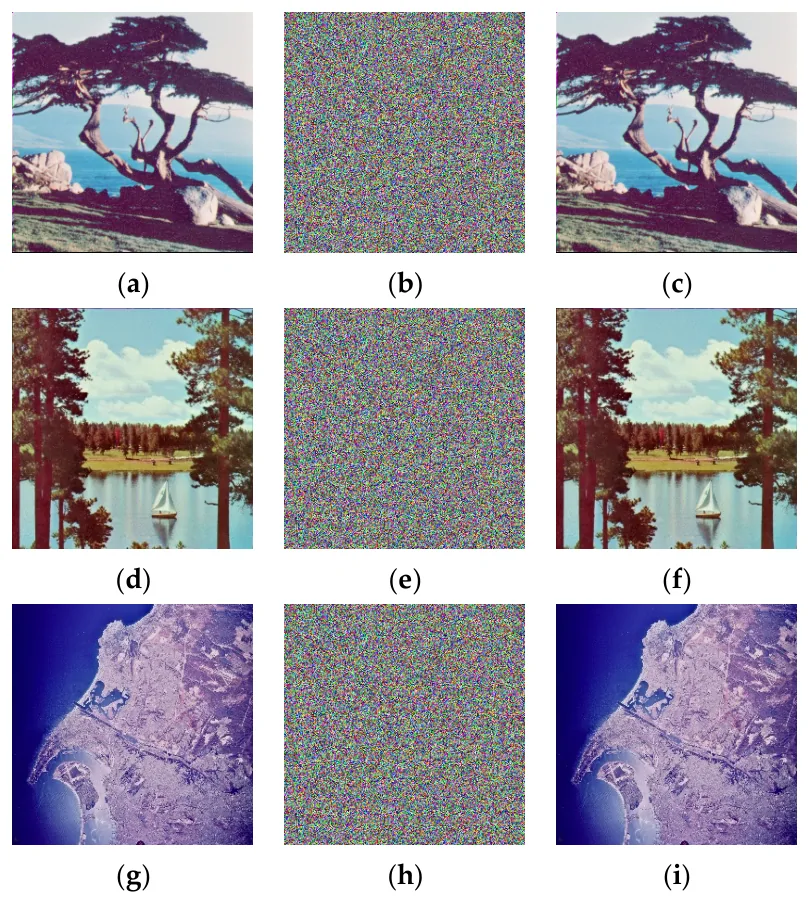
Encryption and decryption tests: Plain images are (**a**) 4.1.06, (**d**) 4.2.06, and (**g**) 2.2.01; (**b**), (**e**), and (**h**) represent the corresponding cipher images for (**a**), (**d**), and (**g**), respectively; (**c**), (**f**), and (**i**) represent the decrypted and restored images for the aforementioned plain images, respectively.

A secure image-encryption algorithm should transform natural images into ciphertext that reveals no meaningful visual information. To examine the visual behavior of the proposed scheme in both encryption and decryption, three color images with different resolutions were selected from the USC-SIPI dataset: 4.1.06 (256 × 256 × 3), 4.2.06 (512 × 512 × 3), and 2.2.01 (1024 × 1024 × 3). The corresponding results are shown in Figure 14.

After encryption, all three plaintext images are converted into visually noise-like ciphertext, whose structural content and texture patterns are effectively obscured. Moreover, the correct secret key is used, and the decryption process reconstructs the original images without visible artifacts. These results indicate that the proposed scheme provides robust visual concealment while preserving reversibility.

### 6.2. Key Space Analysis

To resist brute-force attacks, the key space of an image-encryption scheme is generally expected to exceed 2^100^ [49]. The proposed secret key consists of six independent real-valued parameters, namely $\left\{x_{0},y_{0},a,b,\mu ,\beta \right\}$. All experiments were conducted on Windows 10 using MATLAB R2021b with the IEEE 754 double-precision floating-point format. Following the conventional estimate used in chaotic image-encryption studies, a decimal precision of approximately 10^−15^ is adopted. The nominal key space is consequently estimated as (10^15^)^6^ = 10^90^. This key space is substantially larger than 2^100^, indicating that the proposed algorithm provides a sufficiently large key space against brute-force attacks.

### 6.3. Histogram Analysis

Histogram analysis is a standard tool for assessing resistance to statistical attacks.

**Figure 15:**
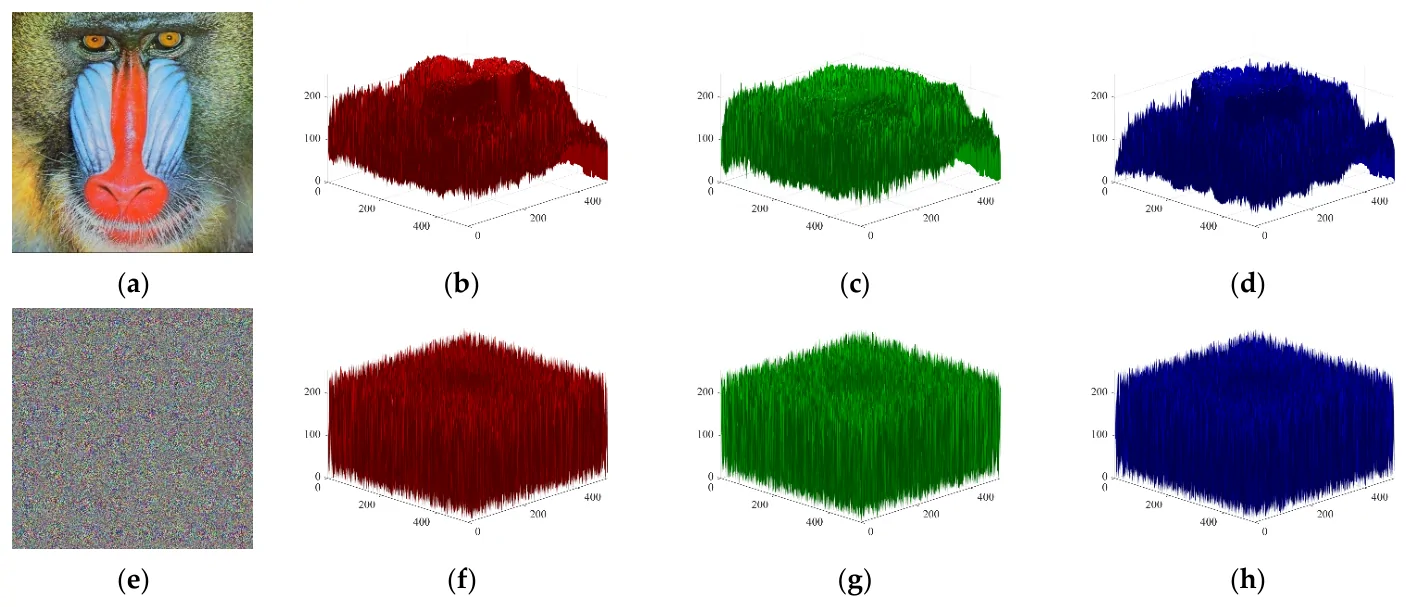
4.2.03 Histogram analysis of images: (**a**) 4.2.03; (**b**) plaintext channel R; (**c**) plaintext channel G; (**d**) plaintext channel B; (**e**) 4.2.03 ciphertext image (**f**) ciphertext channel R; (**g**) ciphertext channel G; (**h**) ciphertext channel B.

As shown in Figure 15, the plaintext histograms present obvious fluctuations, whereas the ciphertext histograms produced by the proposed algorithm are substantially flattened and approach a uniform distribution. These findings indicate that the proposed permutation–diffusion process effectively suppresses statistical leakage in pixel values and improves the randomness of the ciphertext, thereby strengthening resistance to frequency-based statistical attacks.

### 6.4. Information Entropy and Local Shannon Entropy Analysis

Information entropy is a widely used quantitative indicator for measuring the randomness and unpredictability of an information source.

The entropy results of the proposed scheme are reported in Table 3. As expected, the cipher images consistently achieve entropy values above 7.9968, with the best case reaching 7.9998, which is very close to the theoretical upper bound of 8 for 8-bit images. These results indicate that the proposed scheme effectively suppresses the statistical characteristics of the plaintext and promotes an approximately uniform pixel-value distribution, thereby improving resistance to entropy-based statistical analysis.

Table 4 further compares the entropy values with those of several representative encryption algorithms [25,50,51,52]. The proposed scheme achieves comparable or greater entropy across the tested images, suggesting improved ciphertext randomness relative to the competing methods.

Following the method proposed by Wu et al. [53], the local Shannon entropy is computed over *k* = 30 non-overlapping 44 × 44 image blocks *T_B_* = 1936, and the 95% ideality confidence interval [7.901901, 7.903037] (centered at 7.9025) is adopted as the randomness criterion. As shown in Table 5, the entropy of each channel after encryption closely approaches the ideal center, indicating that the proposed scheme satisfies the local randomness criterion at the 95% confidence level.

### 6.5. Correlation Analysis

Digital images typically exhibit inherent redundancy, which leads to pronounced statistical correlation between spatially adjacent pixels. To evaluate the proposed scheme in terms of correlation suppression, adjacent-pixel correlation tests were conducted on images before and after encryption. In the experiments, 10,000 pairs of neighboring pixels were randomly selected along the horizontal, vertical, and diagonal directions. The correlation coefficient $\gamma_{xy}$ is defined as follows:

(16) $$\gamma_{xy}=\frac{cov\left(x,y\right)}{\sqrt{D\left(x\right)}\sqrt{D\left(y\right)}},$$

(17) $$cov(x,y)=\frac{1}{T}\sum_{i=1}^{T}\left(x_{i}-\bar{x})(y_{i}-\bar{y}\right),$$

Here, *x* and *y* denote two neighboring pixel sequences, and $x_{i}$ and $y_{i}$ are the gray-level values of the *i*-th selected pixel pair. cov (⋅) denotes the covariance, and D(⋅) denotes the variance of the pixel values. In addition, $\bar{x}$ and $\bar{y}$ represent the arithmetic means of the two sequences, and *T* is the total number of randomly selected pixel pairs used in the calculation.

**Figure 16:**
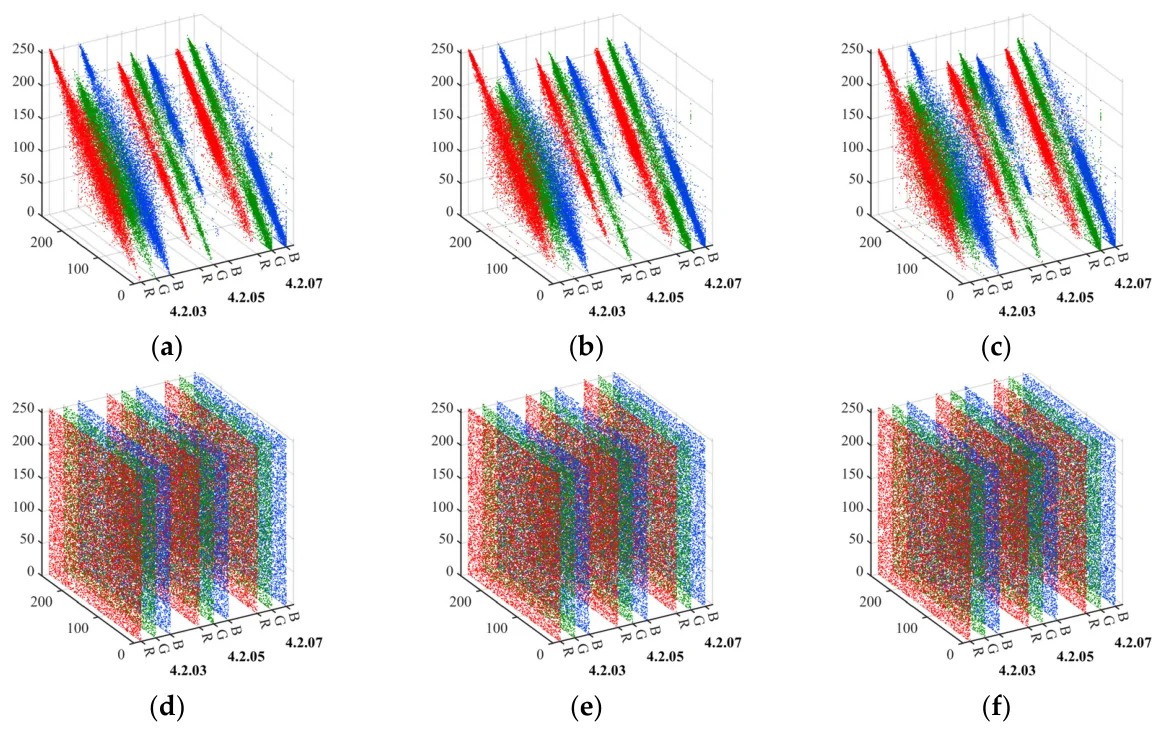
Image correlation analysis: (**a**–**c**) represent the adjacent pixel correlations of the plain image in three directions; (**d**–**f**) represent the adjacent pixel correlations of the cipher image in three directions.

Figure 16 provides an intuitive visualization of adjacent-pixel correlation. Figure 16a–c show pixel-pair scatter distributions of plain images 4.2.03, 4.2.05, and 4.2.07 along horizontal, vertical, and diagonal directions; Figure 16d–f show the corresponding cipher-image plots. In each panel, RGB channels of the three images are grouped along the *x*-axis for direct comparison. Plain-image pairs cluster tightly along the diagonal in every channel, indicating strong adjacent-pixel correlation. Cipher-image pairs disperse uniformly across the plane for all channels and images, confirming that neighborhood dependencies are removed regardless of content.

Table 6 further reports the quantitative correlation coefficients before and after encryption. The correlation coefficients of the plain image are close to 1 in all three directions, whereas the ciphertext coefficients decrease sharply and approach 0. Table 7 additionally compares these results with those of other encryption schemes, and the proposed method achieves better performance in most cases, highlighting its effectiveness in reducing adjacent-pixel correlation.

### 6.6. Differential Attack Analysis

Differential cryptanalysis attempts to infer secret information by analyzing how small changes in the plaintext affect the resulting ciphertext. To quantitatively evaluate resistance to such attacks, two standard metrics are used: the normalized pixel change rate (NPCR) and the unified average changing intensity (UACI). Their definitions are given as follows:

(18) $$NPCR=\frac{\sum_{x,y}\delta \left(x,y\right)}{W\times H}\times 100\%,$$

(19) $$UACI=\frac{1}{W\times H}\sum_{x,y}\frac{\left|C_{1}\left(x,y\right)-C_{2}\left(x,y\right)\right|}{L-1}\times 100\%,$$

Here, δ(x,y) is a decision function: if $C_{1}\left(x,y\right){\neq C}_{2}\left(x,y\right)$, then δ(x,y)=1; otherwise, δ(x,y)=0. *W* and *H* denote the image width and height, respectively, and *L* is the gray-level range. In general, a scheme with strong resistance to differential attacks is expected to achieve NPCR and UACI values close to the theoretical reference values of 99.6094% and 33.4635%, respectively [54].

To evaluate differential robustness, the NPCR and UACI are computed between the original ciphertext *C*_1_ and a second ciphertext *C*_2_ obtained by changing only one pixel in the plaintext. As reported in Table 8, the measured values for all test images are close to the theoretical references. These findings indicate that a single-pixel perturbation in the plaintext induces widespread changes in the ciphertext, reflecting an avalanche effect. Consequently, the statistical relationship between plaintext differences and ciphertext differences is substantially weakened, which increases the difficulty of recovering secret information via differential analysis and supports the resistance of the proposed scheme to differential attacks.

### 6.7. Key Sensitivity Analysis

**Figure 17:**
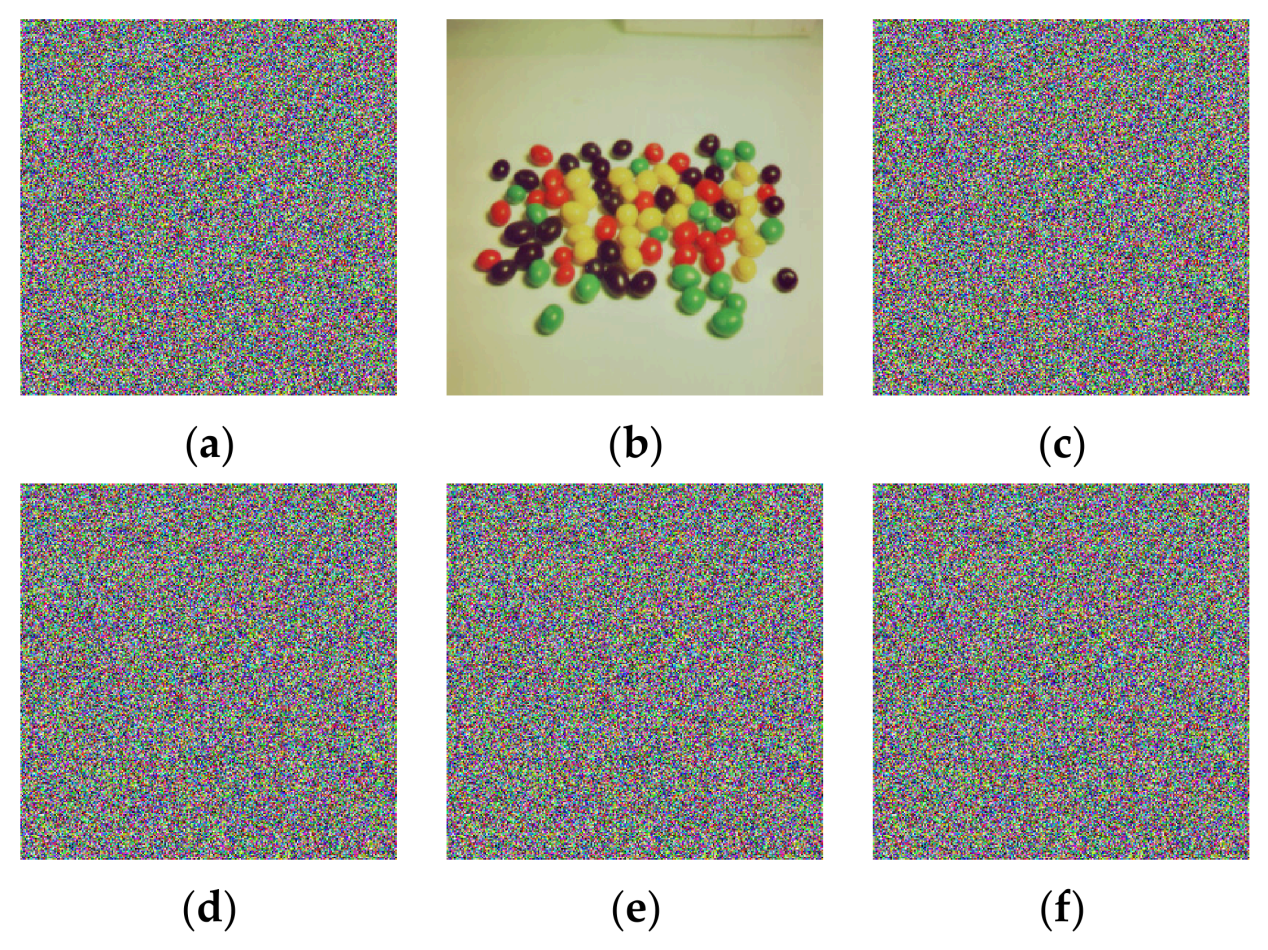
Key sensitivity test results: (**a**) cipher image encrypted using *Key*_0_ keys; (**b**) recovered image decrypted using *Key*_0_ keys; (**c**–**f**) images decrypted using *Key*_1_, *Key*_2_, *Key*_3_, and *Key*_4_.

A secure cryptographic system should exhibit high sensitivity to even the slightest modification of the key. The sensitivity of the secret key is verified as follows. The benchmark key is set to ${Key}_{0}=\{x_{0}=0.6,y_{0}=0.5,a=8,b=8,\mu =12,\beta =10\}$. To assess key sensitivity, each component of the secret key is subjected to a perturbation of 10^−14^, generating the following modified keys: *Key*_1_ alters only *a* to $8+10^{-14}$, *Key*_2_ alters only *b* to $8+10^{-14}$, *Key*_3_ alters only *x*_0_ to $0.6+10^{-14}$, and *Key*_4_ alters only *y*_0_ to $0.5+10^{-14}$. The correct key *Key*_0_ and the modified keys *Key*_1_, *Key*_2_, *Key*_3_, and *Key*_4_ are subsequently used to decrypt the cipher images. Figure 17 presents the results of the key sensitivity test, which demonstrate that only the correct key can accurately recover the plain image. Even when the secret key is altered by merely 10^−14^, they fail to reconstruct the plain image. Consequently, the proposed encryption scheme is highly sensitive to the key.

### 6.8. Noise and Cropping Attacks

In practical transmission scenarios, encrypted image data may be corrupted by channel noise or partially lost because of packet drops and truncation. To evaluate the robustness of the proposed method, a set of experiments was conducted on image 4.2.05 to examine recovery performance under different levels of noise contamination and ciphertext cropping.

**Figure 18:**
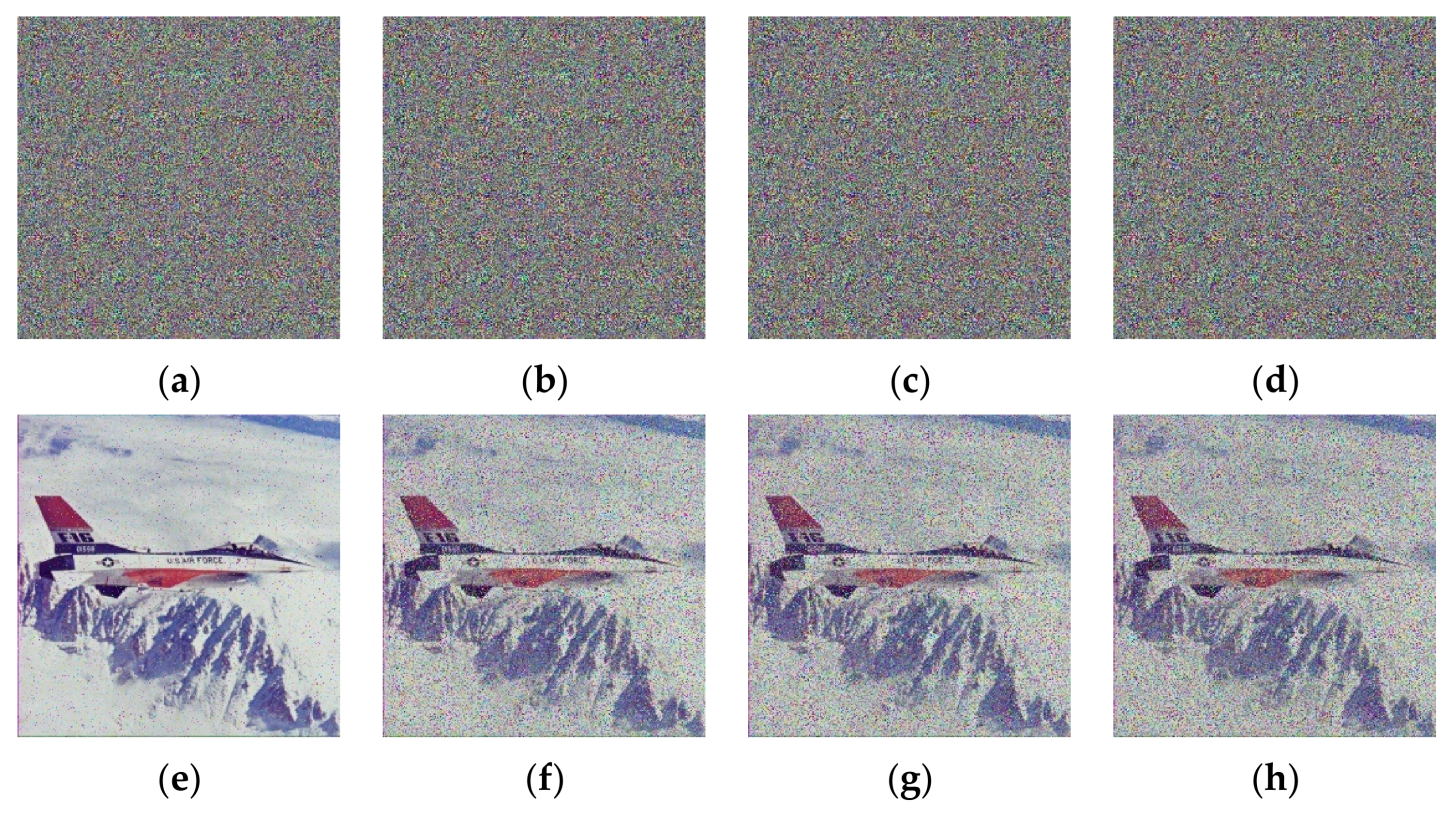
Robustness against attack experimental results: (**a**) Cipher image with 0.5% salt-and-pepper noise added; (**b**) Cipher image with 5% salt-and-pepper noise added; (**c**) cipher image with 6% salt-and-pepper noise added; (**d**) cipher image with 8% salt-and-pepper noise added; (**e**) decrypted and restored image corresponding to (**a**); (**f**) decrypted and restored image corresponding to (**b**); (**g**) decrypted and restored image corresponding to (**c**); (**h**) decrypted and restored image corresponding to (**d**).

**Noise contamination.** In the noise-resistance test, salt-and-pepper noise with densities of 0.5%, 5%, 6%, and 8% was injected into the cipher images. The corresponding decryption results are shown in Figure 18. As expected, the visual quality of the decrypted images gradually decreases as the noise density increases, with blurring and artifacts becoming more apparent. Nevertheless, even at a noise level of 8%, the main structural content of the recovered image remains largely intact and visually recognizable. This suggests that the proposed scheme maintains good tolerance to relatively impulsive noise, which is beneficial for deployment in complex communication environments.

**Figure 19:**
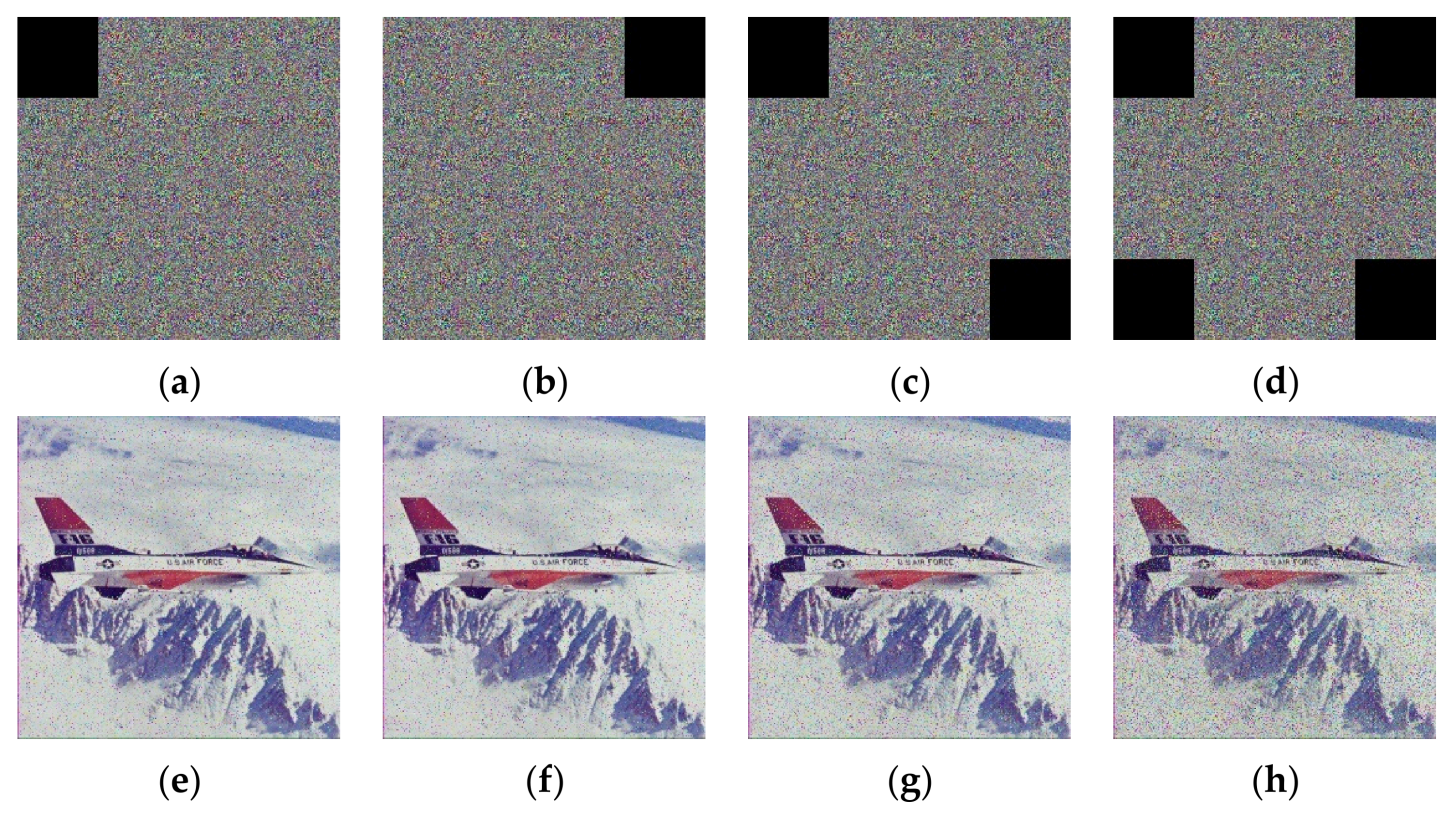
Crop attack experimental results: (**a**) Cipher image with 128 × 128 × 3 pixels missing from the top-left corner; (**b**) cipher image with 128 × 128 × 3 pixels missing from the top-right corner; (**c**) cipher image with 128 × 128 × 3 pixels missing from both the top-left and bottom-right corners; (**d**) cipher image with 128 × 128 × 3 pixels missing from all four corners; (**e**) decrypted and restored image corresponding to (**a**); (**f**) decrypted and restored image corresponding to (**b**); (**g**) decrypted and restored image corresponding to (**c**); (**h**) decrypted and restored image corresponding to (**d**).

**Ciphertext cropping.** For the cropping attack, four cropping cases at different locations were designed, and in each case, a (128 × 128) block was removed from the ciphertext. The results are presented in Figure 19. Although the decrypted images contain noticeable artifacts because of missing ciphertext data, their principal contours, textures, and overall visual content remain identifiable. These observations indicate that the proposed encryption scheme exhibits favorable robustness under partial data loss and can still support meaningful reconstruction when the ciphertext is truncated.

### 6.9. Chosen-Plaintext Attack Analysis

Attackers may deliberately select special images with extremely low information entropy, such as completely white or completely black inputs, to probe the keystream or infer diffusion rules, which is a typical chosen plaintext setting. To examine robustness under this scenario, we encrypted fully white and fully black images.

**Figure 20:**
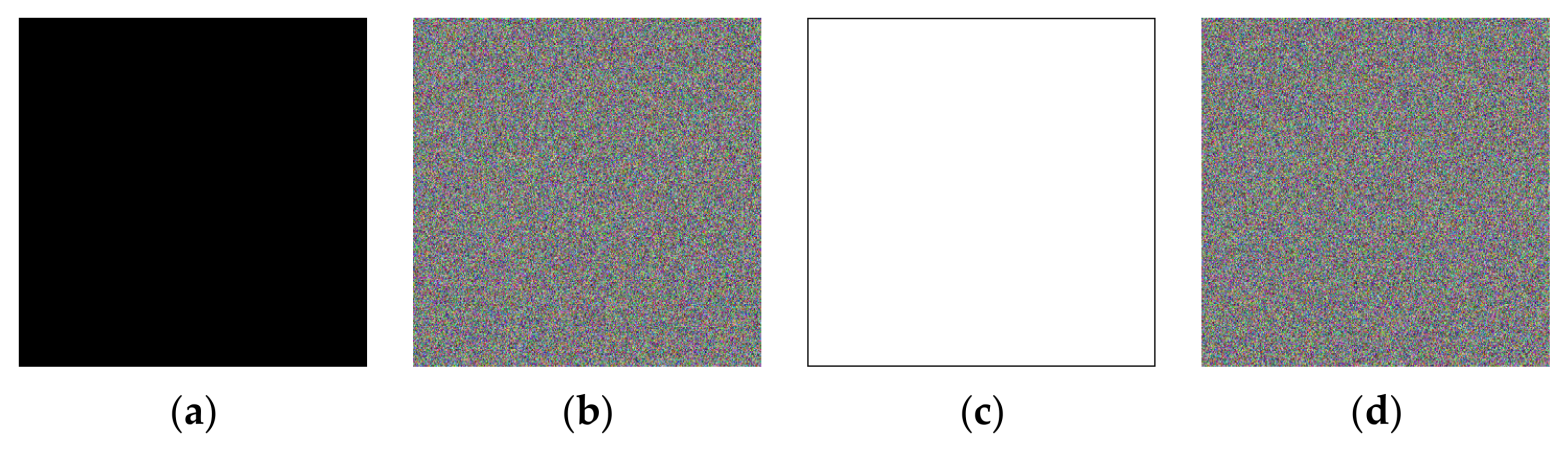
Simulation results for specific images: (**a**) all-black image; (**b**) cipher image after encryption of the all-black image; (**c**) all-white image; (**d**) cipher image after encryption of the all-white image.

As shown in Figure 20, although these plaintexts contain only a single pixel value and therefore exhibit maximal redundancy, the resulting ciphertexts display a visually uniform, noise-like appearance with no discernible structure. This suggests that the proposed scheme does not leak exploitable patterns under low-entropy plaintexts and provides good resistance against chosen-plaintext attacks.

### 6.10. Computational Complexity Analysis

Let the image size be *m* × *n*. The key-sequence generation stage produces four pseudo-random streams via the proposed 2D hyperchaotic map with complexity *O*(4*mn*). The permutation stage traverses all *mn* pixels along a doubly linked list with an adaptive step bounded by $\frac{\sqrt{mn}}{2}$, giving *O*(*mn* $\times \sqrt{mn}$). The diffusion stage performs forward and backward cyclic-shift XOR sweeps at O(2*mn*). The overall complexity is therefore O(*mn* $\times \sqrt{mn}\;+\;$6*mn*).

Consistent with this analysis, Table 9 reports the encryption time (EH) and throughput (ETH) of the proposed algorithm against several recent schemes. For a 512 × 512 × 3 color image, the proposed method requires 1.7329 s, which is slightly longer than that of some competing methods because the adaptive-step linked-list permutation scales superlinearly with image size. Nevertheless, its throughput of 0.4328 MB/s remains acceptable for full-color image encryption. This additional computational cost represents a trade-off for enhanced positional disruption and resistance to statistical attacks.

## 7. Conclusions

By integrating the strongly coupled valley structure of the Rosenbrock function with the multimodal oscillatory characteristics of the Schwefel function, we propose a novel two-dimensional RS hyperchaotic map (2D-RS). Numerical simulations show that 2D-RS exhibits stable hyperchaotic behavior and favorable ergodicity over a broad parameter range. Building on this map, we design a dynamic bidirectional oscillatory permutation strategy and a state-dependent adaptive dynamic cyclic-shift diffusion mechanism to construct an image-encryption scheme. Security analyses and performance evaluations demonstrate that the proposed scheme provides favorable encryption performance and robust resistance to attacks.

The current implementation targets lossless images; lossy compression may disrupt pixel-index correspondence, diffusion synchronization, and decryption accuracy. Future work will investigate compression-aware encryption, compressive sensing, and parallel or GPU-based acceleration.

## Figures and Tables

**Table 1:** Existing 2D chaotic maps with control parameters.

Reference	Name	*F* (*x*, *y*)	Parameters
[37]	2D-CICM	$\{\begin{array}{c} x_{n+1}=sin(a\pi^{2}/(cos(b\;arccos(x_{n}))(1-cos(b\;arccos(y_{n})))))\\ y_{n+1}=sin(b\pi^{2}/(cos(b\;arccos(y_{n}))(1-cos(b\;arccos(x_{n}))))) \end{array}$	*a*, *b*
[20]	2D-LCIC	$\{\begin{array}{c} x_{n+1}=sin(a\pi /x_{n}y_{n}(1-x_{n}y_{n}))\\ y_{n+1}=sin(b\pi /((x_{n}+y_{n})(1-{\left(x_{n}+y_{n}\right)}^{2}))) \end{array}$	*a*, *b*
[19]	2D-LRNM	$\{\begin{array}{c} x_{n+1}=sin(\sigma_{1}/(1+x_{n}^{2})+y_{n}+\omega_{1}x_{n}(1-y_{n}))\\ y_{n+1}=sin(y_{n}-\sigma_{2}x_{n}-\sigma_{3}+\omega_{2}y_{n}(1-x_{n})) \end{array}$	$\sigma_{1}$, $\sigma_{2}$, $\sigma_{3}$, $\omega_{1}$, $\omega_{2}$
[38]	2D-SHFM	$\{\begin{array}{c} x_{n+1}=sin(a\pi^{2}/{(x}_{n}y_{n}))\\ y_{n+1}=sin(b\pi^{2}x_{n}(1-y_{n})) \end{array}$	*a*, *b*
[20]	2D-ISCC	$\{\begin{array}{c} x_{n+1}=sin(a\pi sin(x_{n}y_{n}))\\ y_{n+1}=sin(b\pi (x_{n}+y_{n})(1-{\left(x_{n}+y_{n}\right)}^{2})) \end{array}$	*a*, *b*

**Table 2:** NIST test results.

No.	Statistical Test	*p* Value of *x*	S or F (*x*)	*p* Value of *y*	S or F (*y*)
1	Frequency	0.2576	S	0.2846	S
2	Block-frequency	0.1583	S	0.3427	S
3	Cumulative sums	0.7318	S	0.4752	S
4	Runs	0.6867	S	0.2131	S
5	Longest runs of Ones	0.7385	S	0.1165	S
6	Rank	0.0408	S	0.2777	S
7	Discrete Fourier transform test	1.0000	S	0.4506	S
8	Non overlapping template matching	0.0964	S	0.9752	S
9	Overlapping template matching	0.2337	S	0.7955	S
10	Universal statistical	0.9748	S	0.4622	S
11	Approximate entropy	0.4512	S	0.9523	S
12	Random excursions	1.0000	S	1.0000	S
13	Random excursions variant	0.1317	S	0.3110	S
14	Serial	0.1315	S	0.0207	S
15	Linear complexity	0.5308	S	0.1842	S

**Table 3:** Information entropy of cipher images in the R, G, and B individual channels.

Size	Image	Plain R	Plain G	Plain B	Cipher R	Cipher G	Cipher B
1024 × 1024 × 3	2.2.01	7.7575	7.3387	6.9561	7.9998	7.9998	7.9998
1024 × 1024 × 3	2.2.05	7.4445	7.2346	6.5896	7.9998	7.9998	7.9998
1024 × 1024 × 3	2.2.08	7.7229	7.5289	6.8318	7.9998	7.9998	7.9998
512 × 512 × 3	4.2.03	7.7067	7.4744	7.7522	7.9994	7.9993	7.9993
512 × 512 × 3	4.2.05	6.7178	6.7990	6.2138	7.9993	7.9993	7.9993
512 × 512 × 3	4.2.07	7.3388	7.4963	7.0583	7.9993	7.9993	7.9993
256 × 256 × 3	4.1.05	6.4311	6.5389	6.2320	7.9976	7.9968	7.9974
256 × 256 × 3	4.1.06	7.2104	7.4136	6.9207	7.9973	7.9976	7.9976
256 × 256 × 3	4.1.07	5.2626	5.6947	6.5464	7.9972	7.9975	7.9973

**Table 4:** Cipher image information entropy comparison analysis across the R, G, and B channels.

Image	Channel	[25]	[50]	[51]	[52]	Proposed
4.2.03	R	7.9993	7.9992	7.9993	-	7.9994
	G	7.9992	7.9993	7.9993	-	7.9993
	B	7.9993	7.9992	7.9993	-	7.9993
4.2.07	R	7.9993	7.9991	7.9992	7.9993	7.9993
	G	7.9994	7.9992	7.9993	7.9993	7.9993
	B	7.9992	7.9992	7.9993	7.9992	7.9993

**Table 5:** Local entropy of cipher images in the R, G, and B individual channels.

Image	Plain R	Plain G	Plain B	Cipher R	Cipher G	Cipher B
2.2.01	6.1976	5.5680	5.4120	7.9020	7.9003	7.9027
2.2.05	6.7882	6.4635	5.7994	7.8997	7.9047	7.9025
2.2.08	6.4940	6.1577	5.3923	7.9028	7.9010	7.9039
4.2.03	6.5207	6.9240	6.7512	7.9016	7.9029	7.9009
4.2.05	5.1153	5.7048	5.1815	7.9029	7.9067	7.9028
4.2.07	6.0375	6.0249	5.9805	7.9029	7.9028	7.9020
Mean	6.1922	6.1405	5.7528	7.9020	7.9031	7.9025

**Table 6:** Correlation between adjacent pixels in plain images across three directions.

Image	Direction	Plain R	Plain G	Plain B	Cipher R	Cipher G	Cipher B
2.2.01	Horizontal	0.926506	0.920748	0.909147	0.003877	−0.003882	−0.005246
	Vertical	0.920383	0.914158	0.906562	−0.000479	0.002018	0.002326
	Diagonal	0.904060	0.891374	0.884262	−0.008652	0.005260	−0.000334
2.2.05	Horizontal	0.938527	0.944266	0.897832	−0.006536	−0.005707	−0.003392
	Vertical	0.929513	0.924286	0.878533	0.000296	0.004099	0.001342
	Diagonal	0.908868	0.915538	0.875350	0.004142	−0.003659	0.000972
2.2.08	Horizontal	0.922927	0.920235	0.900228	0.003824	−0.003666	−0.003271
	Vertical	0.922631	0.912370	0.891840	−0.003303	0.000658	0.006389
	Diagonal	0.904331	0.895095	0.871136	−0.004262	0.004048	−0.005116
4.2.03	Horizontal	0.920144	0.871085	0.903918	−0.000802	0.005571	−0.000525
	Vertical	0.871106	0.753188	0.879148	0.000281	−0.000751	0.001744
	Diagonal	0.858287	0.744541	0.845235	−0.00057	0.000688	0.001098
4.2.05	Horizontal	0.974016	0.955423	0.964890	0.001767	0.000190	0.002774
	Vertical	0.959348	0.964018	0.935032	−0.002039	−0.006399	−0.000457
	Diagonal	0.934451	0.938564	0.913482	0.003709	−0.000335	−0.002053
4.2.07	Horizontal	0.965928	0.980581	0.968250	−0.002989	0.001654	−0.00064
	Vertical	0.965683	0.980843	0.968139	0.003457	0.000957	−0.000713
	Diagonal	0.961562	0.965546	0.948582	−0.000334	−0.000205	0.000264
4.1.06	Horizontal	0.957655	0.969344	0.960901	0.002696	−0.000769	0.009187
	Vertical	0.936754	0.942640	0.943474	0.002895	−0.007363	0.004369
	Diagonal	0.917956	0.931956	0.927970	0.003832	−0.002387	−0.005014
4.1.07	Horizontal	0.975149	0.973212	0.988038	0.000313	−0.008739	−0.005658
	Vertical	0.978002	0.980417	0.988020	0.001538	0.006290	0.001060
	Diagonal	0.958514	0.960670	0.980307	−0.004597	0.000893	0.000733
4.1.05	Horizontal	0.965973	0.982242	0.981483	−0.000318	−0.001982	−0.001167
	Vertical	0.935828	0.952571	0.974160	−0.004442	−0.001128	0.002311
	Diagonal	0.914885	0.933288	0.963357	0.001606	−0.003309	0.007272

**Table 7:** Correlation analysis of cipher images across the R, G, and B channels.

Images	Dir	Channel	[25]	[50]	[51]	[52]	Proposed
4.2.03	H	R	−0.0012	−0.0077	−0.00006	-	−0.0008
		G	0.0005	0.0038	0.0005	-	0.005571
		B	−0.0001	−0.008	−0.0003	-	−0.00053
	V	R	0.0003	0.0056	0.0005	-	0.000281
		G	0.0006	−0.0029	0.0006	-	−0.00075
		B	−0.0003	−0.0028	−0.00003	-	0.001744
	D	R	0.0048	0.0016	−0.0013	-	−0.00057
		G	−0.0062	−0.0017	−0.0005	-	0.000688
		B	−0.0076	0.0001	0.0008	-	0.001098
4.2.07	H	R	0.0067	0.0057	0.0014	0. 0027	−0.00299
		G	−0.0062	−0.0098	0.0008	0.0017	0.001654
		B	0.0048	0.0068	0.0012	0.0014	−0.00064
	V	R	0.0036	0.0036	0.0025	0.0016	0.003457
		G	0.0037	0.0037	0.0019	0.0001	0.000957
		B	0.0043	0.0043	−0.0003	0.0005	−0.00071
	D	R	−0.0073	−0.0058	−0.00009	0.0015	−0.00033
		G	−0.0040	−0.0012	−0.0034	0.0004	−0.00021
		B	0.0041	−0.0022	0.0041	0.0003	0.000264

**Table 8:** NPCR (%) and UACI (%) metrics for cipher images.

Image	NPCR	UACI
2.2.01	99.6083	33.4634
2.2.05	99.6061	33.4731
2.2.08	99.6132	33.4472
4.2.03	99.6157	33.4985
4.2.05	99.6001	33.4655
4.2.07	99.6175	33.4347
4.1.05	99.5860	33.4492
4.1.06	99.6109	33.4908
4.1.07	99.6109	33.4923
Average	99.6076	33.4683

**Table 9:** Encryption time (seconds) and encryption throughput for different sizes.

Reference	Size	EH	ETH
[25]	512 × 512 × 3	0.8569	0.8752
[50]	256 × 256 × 3	0.4262	-
	512 × 512 × 3	0.9220	-
[51]	256 × 256	0.0794	0.7872
	512 × 512 × 3	0.9447	0.2646
[52]	256 × 256 × 3	0.077	-
	512 × 512 × 3	0.281	-
Proposed	256 × 256	0.1792	0.3488
	256 × 256 × 3	0.4789	0.3915
	512 × 512 × 3	1.7329	0.4328

## Data Availability

Data will be made available on request.
